# Bidirectional promoters exhibit characteristic chromatin modification signature associated with transcription elongation in both sense and antisense directions

**DOI:** 10.1186/s12864-018-4697-7

**Published:** 2018-05-02

**Authors:** Rahul Kumar Jangid, Ashwin Kelkar, Vijaykumar Yogesh Muley, Sanjeev Galande

**Affiliations:** Centre of Excellence in Epigenetics, Indian Institute of Science Education and Research, Dr Homi Bhabha Road, Pune, 411008 India

**Keywords:** Bidirectional promoters, Transcription regulation, Histone modifications, Chromatin landscape

## Abstract

**Background:**

In contrast to unidirectional promoters wherein antisense transcription results in short transcripts which are rapidly degraded, bidirectional promoters produce mature transcripts in both sense and antisense orientation. To understand the molecular mechanism of how productive bidirectional transcription is regulated, we focused on delineating the chromatin signature of bidirectional promoters.

**Results:**

We report generation and utility of a reporter system that enables simultaneous scoring of transcriptional activity in opposite directions. Testing of putative bidirectional promoters in this system demonstrates no measurable bias towards any one direction of transcription. We analyzed the NUP26L-PIH1D3 bidirectional gene pair during Retinoic acid mediated differentiation of embryonic carcinoma cells. In their native context, we observed that the chromatin landscape at and around the transcription regulatory region between the pair of bidirectional genes is modulated in concordance with transcriptional activity of each gene in the pair. We then extended this analysis to 974 bidirectional gene pairs in two different cell lines, H1 human embryonic stem cells and CD4 positive T cells using publicly available ChIP-Seq and RNA-Seq data. Bidirectional gene pairs were classified based on the intergenic distance separating the two TSS of the transcripts analyzed as well as the relative expression of each transcript in a bidirectional gene pair. We report that for the entire range of intergenic distance separating bidirectional genes, the expression profile of such genes (symmetric or asymmetric) matches the histone modification profile of marks associated with active transcription initiation and elongation.

**Conclusions:**

We demonstrate unique distribution of histone modification marks that correlate robustly with the transcription status of genes regulated by bidirectional promoters. These findings strongly imply that occurrence of these marks might signal the transcription machinery to drive maturation of antisense transcription from the bidirectional promoters.

**Electronic supplementary material:**

The online version of this article (10.1186/s12864-018-4697-7) contains supplementary material, which is available to authorized users.

## Background

In the human genome a substantial portion of genes (10%) are arranged in a head-to-head manner, referred to as bidirectional genes [1]. A “bidirectional gene pair” is defined as two adjacent genes whose coding sequences are located on opposite strands of DNA with transcription start sites (TSSs) not more than 1 Kilo base pairs (Kb) apart. The intergenic region between two adjacent TSSs is commonly designated as a putative “bidirectional promoter”. The abundance of bidirectional promoters is a common feature among all mammalian genomes, suggesting a role of evolutionary pressure in the conservation of such structure of gene organization [2, 3]. It has been shown that bidirectional promoters regulate transcription of gene pairs wherein the transcript levels from each gene need to be coordinately regulated [2]. Various studies have analyzed the presence of promoter elements in the bidirectional promoters and showed that the occurrence of the TATA box is underrepresented in bidirectional promoters and only 9% bidirectional promoters harbor the TATA box as compared to 29% of the unidirectional promoters [4]. Furthermore, 77% of bidirectional promoters harbor CpG islands as compared to 38% of unidirectional promoters [5, 6]. However, significance of such differential occurrence of these promoter elements within bidirectional and unidirectional promoters is not known. A recent study in *Saccharomyces cerevisiae* revealed that cryptic transcripts arise from divergent transcription from well-defined gene promoters, which suggest that bidirectional transcription is an inherent property of eukaryotic promoters [7]. These cryptic transcripts are immediately targeted for degradation by the Nrd1–exosome– TRAMP complexes [8]. A few studies have shown that all the mammalian active promoters have ability to transcribe in both sense and antisense direction but productive transcription occurs only in the sense orientation and antisense transcripts upstream to the promoter are targeted for immediate degradation [9–12]. However, the ability to produce productive sense and antisense transcripts is unique to bidirectional promoters and the reasons for the same remain unclear. The presence of different sequence elements on bidirectional promoters as compared to unidirectional promoters has not been able to explain the productive bidirectional transcription from these promoters. An additional mechanism affecting transcription includes alteration in chromatin architecture that is typically accompanied by signature histone modifications that are characteristic of transcription initiation and elongation [13–15]. Histone modifications regulate the transcription by facilitating or restricting the binding of transcription activator or repressor complexes [16]. Histone modification marks are subject to change depending on transcriptional status of the genes in question, which makes them very attractive candidates for regulating bidirectional transcription. In this study we have analyzed the unique chromatin signature of bidirectional promoters and its correlation with productive transcription in both sense and antisense orientation.

To monitor bidirectional transcription, we designed an experimental system with two reporter genes arranged in a head to head orientation without any promoter element in between them as described recently [17]. We then cloned eight representative bidirectional promoters in sense and antisense orientations and observed their efficient transcription in both orientations. We selected one such intergenic region containing a putative bidirectional promoter and performed ChIP analysis to study the occupancy of the histone marks associated with active transcription. Most strikingly, we observed bimodal distribution of active transcription associated histone marks in the vicinity of the TSSs.

To verify whether the observed bimodal pattern holds true across most, if not all, bidirectional promoters, we further analyzed publicly available datasets for histone modifications associated with active transcription initiation and successful transcription elongation. Histone modification profiles from two cell types were analyzed to confirm whether these profiles were cell type specific or not. We analyzed Histone 3 Lysine K tri-methylation (H3K4me3), Histone 3 lysine 27 acetylation (H3K27ac) near the TSS of 974 bidirectional gene pairs, and Histone 3 lysine 36, lysine 79 methylation (H3K36me3, H3K79me1) in the gene bodies of the 974 pairs in H1 human embryonic stem (ES) cells and human CD4^+^ T cells. We categorized the bidirectional genes based on the intergenic distance that separates them, in terms of number of nucleosomes that can be accommodated between the two TSS. Such analysis revealed that histone modifications that mark actively transcribing promoters are coincident with transcriptional status of each gene in a bidirectional gene pair.

For pairs wherein the expression is asymmetric, i.e., each gene in a pair is expressed at significantly altered level relative to its partner, a significant bias in H3K4me3 enrichment that mirrors the transcription profile was observed in both the datasets tested. Interestingly, H3K27ac does not show this correlation in both cell types and the correlation is markedly higher in H1 ES cells. In the case of symmetrically expressed bidirectional gene pairs, the histone enrichment profile for H3K4me3 is bimodal and this is never observed in any of the unidirectional genes that were used as a control set of genes. Thus, the bimodal distribution of histone modification marks that strongly correlates with the transcription status of genes presumably constitutes a chromatin signature unique to bidirectional promoters.

## Methods

### Antibodies

Normal rabbit IgG (12–370) and normal mouse IgG (12–371) were purchased from Millipore/Upstate. H3K79me3 (17–10,130), H3K36me3 (17–10,032), H3K27me1 (17–643), H3K4me3 (07–473), Acetyl-Histone H4 (17–630) and H3K9ac (17–658) antibodies were procured from Millipore. All of the above antibodies were used for ChIP analysis at 5 μg antibody per 30 μg chromatin. Oct-3/4 (sc-8628) and Sox2 (sc-17,319) antibodies were procured from Santa Cruz Biotechnology. Anti-Nanog (AF1997) was purchased from R&D Systems. Anti-H3 (ab1791) was procured from Abcam. All of the above antibodies were used for immunoblot analysis at 1:1000 dilution.

### Cell culture, microscopy and flow cytometry analysis

HEK-293 (ECACC Catalog number 85120602) and Jurkat (ECACC Catalog number 88042803) were obtained from the ECACC (European Collection of Authenticated Cell Cultures) repository. HEK293 cells and Jurkat cells were grown in DMEM and RPMI 1640 respectively. For both cells media was supplemented with 10% fetal bovine serum and Penicillin/Streptomycin, at 37 °C under 5% CO_2_ atmosphere. For transfections, HEK-293 T cells were grown up to 60% confluency in 6 well culture plates at 37 °C in DMEM (Gibco) supplemented with 10% fetal bovine serum (FBS) (Invitrogen) and penicillin/streptomycin, under 5% CO_2_ atmosphere. Cells were transfected using Lipofectamine 2000 as per manufacturer’s instructions (Invitrogen). The medium was supplemented with 10% FBS 6 h post-transfection. The cells were allowed to grow for 48 h post-transfection before being imaged by confocal microscopy and flow cytometry analysis. For flow cytometry, cells were washed twice with ice cold PBS, followed by fixing with 3.7% paraformaldehyde. Cells were then acquired using FACS CANTO II flow cytometer (Becton Dickinson).

### RNA isolation and cDNA synthesis

Total RNA was isolated using TRIzol (Invitrogen). Five hundred ng of total RNA was reverse transcribed using ImProm-II™ Reverse Transcription System (Promega). Quantitative real-time PCRs were performed using SYBR green PCR master mix (Applied Biosystems), with annealing and extension of primers at 60 °C. Fold changes were calculated using the formula; Fold change = 2^-(ΔΔCt)^.

### Chromatin immunoprecipitation (ChIP) assay

ChIP assay was performed as described [18]. For quantification of enrichment, the efficiency of chromatin immunoprecipitation of particular genomic locus was calculated from quantitative PCR (qPCR) data and reported as a percentage of starting material: % (ChIP/ Total input) which was calculated using the following formula:

$$ \%\left(\mathrm{ChIP}/\mathrm{Total}\ \mathrm{input}\right)={2}^{\wedge}\left[\left(\mathrm{Ct}\left(\mathrm{x}\%\mathrm{input}\right)\hbox{-} \log \left(\mathrm{x}\%\right)/\log 2\right)-\mathrm{Ct}\left(\mathrm{ChIP}\right)\right]\times 100\% $$

The recovery is the % (ChIP/ Total input). Relative occupancy was calculated as a ratio of specific signal over background:

$$ \mathrm{Occupancy}=\%\mathrm{input}\ \left(\mathrm{specific}\ \mathrm{loci}\right)/\%\mathrm{input}\ \left(\mathrm{background}\ \mathrm{loci}\right) $$

Relative occupancy is then used as a measure of the protein association with a specific locus.

### Differentiation of NT2D1 cells

All-trans-retinoic acid (RA)-induced differentiation of N2D1 cells was performed in 100 mm culture dish. RA was reconstituted at a concentration of 5 mg/ml in DMSO (Sigma) and stored in dark at − 80 °C. For differentiation experiments, NT2D1 cells were harvested using 0.05% Trypsin and resuspended in fresh media. Cells were counted and 2 × 10^6^ cells were seeded in each 100 mm dish. Cells were grown for 24 h prior to addition of RA. Next day, RA was added to a final concentration of 13.7 μM and cells were maintained in RA upto 7 days, with media replacement (containing freshly thawed RA) every day. After 7 days of RA treatment, cells were harvested for ChIP, RNA and protein.

### RNA-seq and ChIP-seq datasets

ENCODE data for H1 ES cells were as follows:

SRR018455 for H3K4me3, SRR029348 for H3K27ac, SRR067953 for H3K79me1, and SRR018454 for H3K36me3 ChIP-Seq respectively.

ENCODE data for CD4 T cells were as follows:

SRR787517 for H3K4Me3 and SRR980415 for H3K27Ac ChIP-Seq respectively.

The RNA-Seq datasets were SRX007165 for H1 ES cells and SRR643766 for CD4 T cells respectively.

### Transcript assembly and abundance estimation

For RNA-Seq, the raw reads were aligned to the hg19 reference assembly using HISAT2 with default options for alignment and the *‘*—no-unal’ option was used to write only the aligned reads to the output file. RSEM [19] was used for transcript assembly and abundance estimation. The list of known transcripts from the hg19 assembly was obtained as a .gtf file from ENCODE. RSEM utilized the nucleotide sequences of transcripts from the GTF file from ENCODE to assemble its own reference sequence. This reference sequence was assembled using the ‘rem-assemble-annotation’ tool. This led to a transcripts.fa file containing the sequences of each transcript from the hg19 genome in the fasta format. Using ‘rsem-estimate-abundance’ command, the expression levels of each transcript in the assembled reference file was estimated using the measure, Transcripts Per Million (TPM).

TPM was used as opposed to the usual Fragments Per Kb Per Million (FPKM) because TPM is a more robust measure of quantitating transcripts even in absence of multiple samples. RSEM abundance is calculated for each transcript in each sample independently. Therefore this abrogates the need of replicates for assigning a significance value to the estimate of transcript abundance. The estimated transcript abundances were stored as text files with ENST IDs that served as unique identification markers for each transcript isoform.

### ChIP-Seq analysis

Histone modifications present in both the data sets were analyzed for comparison across cell types. The raw reads were aligned to the hg19 reference genome assemble using bowtie2 with default options. The alignments for input control and the immunoprecipitated (ChIPed) sample were used to calculate enrichment of histone modifications at all locations in the genome. ‘macs2 callpeak’ was used to identify ‘peaks’ of histone modification enrichment and the output was stored as bedgraph files. A ‘q’ value of 0.05 was used to retain peaks that showed a significant enrichment over Input. *‘*macs2 bdgcmp’ was then used to generate an output file that contained the fold enrichment of that particular histone mark at each location in the genome. The resultant output file was used to plot the enrichment of histone modifications at the desired locations in the genome.

### Plotting histone modification enrichment on bidirectional gene pairs

The list of bidirectional genes was obtained from [20] and each transcript there in was converted to its cognate ENSEMBL ID using the BioMart package in R [21, 22]. After removing transcripts that no longer were considered to be true bonafide transcripts in the hg19 version, transcript pairs with overlapping transcription start sites were eliminated from this list. After confirming that the distance between two TSS of bidirectional genes is less than 1000 bp, 974 gene pairs were used for subsequent analysis. The location of the transcription start and end sites for each transcript were obtained from the VM9 GENCODE database.

### Generation of 4 kb windows flanking the bidirectional promoters

Genomic coordinates for transcription start sites for each partner in a gene pair were used to calculate the mid point between the two TSS for a bidirectional gene pair. If the distance between the two TSSs was an odd number, 1 was added to the total distance before dividing by 2 to find the midpoint between the two TSS. Using the midpoint co-ordinates for each gene pair as a starting point, a 4 Kb window was calculated. These co-ordinates were used for calculating the enrichment of histone modifications. A BED file that contained genomic co-ordinates that lie within each 4 Kb window was calculated using the ‘bedtools chop -stagger’ tool. This generates overlapping genomic co-ordinates that are separated by a specific length and staggered by a specific length, each can be specified independently. The sliding windows used for this analysis were 170 bases in length with a stagger of 75 bases. The tool ‘bedmap -mean’ was used to map the signal of histone enrichment over each interval in the BED file. This plots the mean signal enrichment over input for each histone modification.

### Statistical analysis of histone modifications on bidirectional gene pairs

Genes were classified as symmetric or asymmetric as described above. For each bidirectional gene pair, the midpoint between the two TSS was considered as a reference point for generation of 4 Kb windows as explained above. For each gene pair, histone modifications were quantified on each side of the reference point with overlapping reads discarded from this analysis. In this manner, enrichments of histone modifications were calculated independently for each gene within a bidirectional pair. The cumulative enrichment was calculated using the bedgraph files generated by MACS2 during peak calling. The tool bedmap -sum was used to calculate the cumulative enrichment of signal over input only in cases where the q value of peak calling was lower than 0.05. For asymmetrically expressed gene pairs, the cumulative enrichment of each histone modification for the higher expressing partner gene and lower expressing partner gene from a bidirectional pair was compared with the unpaired t - test. For symmetrically expressed gene pairs, distribution of histone modification enrichment calculated for the ‘upstream’ partner (i.e. upstream of the midpoint) and downstream partner gene (i.e. downstream of the midpoint) was compared with the unpaired t-test. All of the statistical tests were performed using ‘R’ and the resulting data was plotted as violin plots with the ‘p’ value from the t test included.

### Plotting cumulative enrichment of histone modifications

For each histone modification, the enrichment for that modification in each interval in the 4 Kb window was summed over each interval across all bidirectional gene pairs in one gene category. The resultant data was divided by the highest value of enrichment to scale all the values between 0 and 1. This step yielded a normalized profile of histone modifications for each modification in each cell type. Since the size of each window is identical, the windows can be overlaid over each other while keeping the relative locations of the TSS and the intergenic region constant across all bidirectional gene pairs. These are not absolute values and cannot be compared across samples or categories. The plots denote the profile of histone modifications for each set of bidirectional gene pairs with respect to the locations of the transcription start sites for each gene in a pair.

## Results

### Construction of reporter vector to study bidirectional promoter activity

**Fig. 1:**
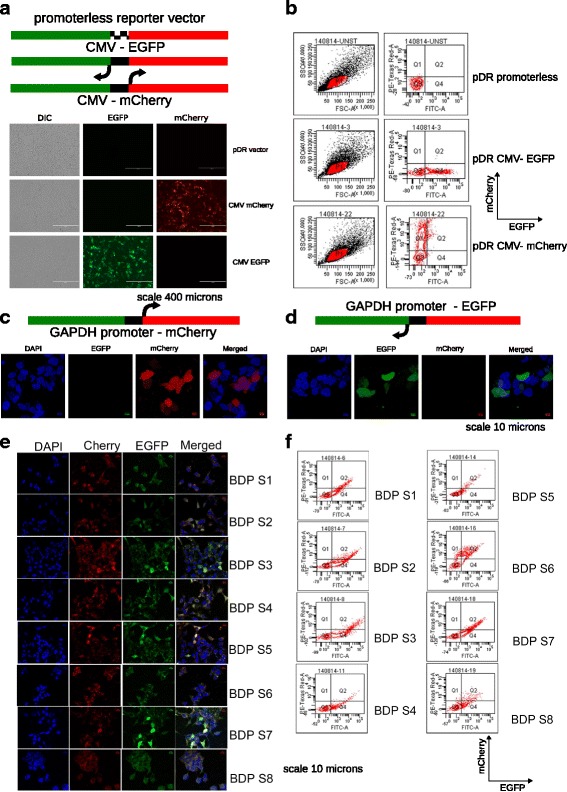
Validation of the pDR reporter vector and bidirectional transcription from human bidirectional promoters. **a** Schematic for orientation of the pDR vector mimicking the organization of bidirectional gene pairs and CMV cloned in sense orientation to either reporter. Images below depict immunofluorescence for pDR vector validation with CMV. Scale bar = 400 μm. **b** Flow cytometry analysis of HEK293T cells transfected by pDR vector and CMV containing pDR vector for validation of promoter activity. Flow cytometry supports microscopy analysis, revealing that there is no transcription from pDR vector alone. CMV promoter clones exhibit transcription of only one of the two reporters and simultaneous reporter transcription of both is virtually undetectable. **c** Schematic shows human GAPDH promoter cloned in pDR in sense orientation to eGFP. Fluorescence imaging for HEK293T cells transfected with this vector is shown below the schematic. **d** The human GAPDH promoter cloned in pDR in sense orientation to mCherry. Images below show fluorescence imaging for HEK293T cells transfected with this vector. **e** Eight intergenic regions from known bidirectional gene pairs cloned in an orientation sense to the eGFP ORF show transcription in either orientation as assayed by eGFP and mCherry fluorescence. The merged images indicate both reporters are transcribed in the transfected cells. Antisense orientation data is presented in Additional file 1: Figure S1. **f** FACS analysis of HEK293 cells transfected with each of the 8 bidirectional promoters. Both reporters are co-expressed in each of bidirectional genes tested

To study bidirectional transcription in vivo we designed a vector system comprising two reporter proteins, namely eGFP and mCherry. These reporter genes are arranged in head-to head orientation and the expression of these reporters can be driven by a common promoter element placed in between the two transcription start sites (Fig. 1a, Additional file 1: Figure S1A). Any genomic region of interest can be cloned into this vector as the intergenic region between these two reporters (Fig. 1a, Additional file 1: Figure S1A). Depending on the expression of one or both the reporters, uni- or bidirectional transcription can be scored. If both reporters are simultaneously expressed only then the intergenic region is considered to exhibit bidirectional promoter activity. Moreover, mutually exclusive expression, depending on the orientation of the cloned sequence would score for transcriptional activity of a unidirectional promoter. A unidirectional promoter would drive the expression of only one of the reporter genes depending on the orientation of cloning while a bidirectional promoter would drive the expression of both the reporters independent of orientation. This vector was generated by modifying the pmCherry-N1 vector such that the constitutive CMV promoter was replaced by eGFP (Additional file 1: Figure S1A). EGFP was cloned in the Ase1 and Nhe1 sites such that the ORF of eGFP would be in opposite orientation to mCherry. Since the CMV promoter is unidirectional we exploited its directional properties to validate the construct (Additional file 1: Figure S1A). The resulting construct, named as pDR vector for plasmid with dual reporters, does not contain any promoter element and therefore does not express any of the reporter genes. If the CMV promoter is cloned in between the mCherry and eGFP, it would drive the expression of either mCherry or GFP depending on the orientation of the cloned promoter (Fig. 1a). To validate this, we transfected pDR vector containing CMV promoter in sense and antisense orientations in HEK 293 T cells. Cells were analyzed 48 h post transfection by microscopy and flow cytometry. We observed that CMV sense and antisense transfected cells specifically express either mCherry or eGFP respectively but never both at the same time (Fig. 1a). Expression of both reporters was virtually undetectable from pDR vector alone (Fig. 1a). Flow cytometry analysis also revealed that CMV promoter drives the expression in directional manner (Fig. 1b). Since CMV promoter is highly specialized viral promoter and represents an extreme case, therefore we also used endogenous GAPDH gene promoter to test if endogenous unidirectional promoters also exhibit directionality bias like the CMV promoter. We cloned the endogenous promoter of human GAPDH gene in pDR vector and observed that upon transfection the endogenous unidirectional GAPDH promoter clones yielded expression of either EGFP or mCherry depending upon the directionality of the promoter (Fig. 1 c & d).

After validating the pDR vector we cloned 8 putative bidirectional promoter regions from previously reported bidirectional gene pairs in the pDR vector in both sense and antisense orientations and transfected them into 293 T cells. Microscopy analysis revealed that all 8 clones express both the reporter proteins in both sense (Fig. 1e) and antisense orientations (Additional file 1: Figure S1B). Details of the genomic regions cloned are provided in Table 1. Additionally, the bidirectional transcription from 8 of these bidirectional promoter containing pDR clones was confirmed by flow cytometry analysis. All of these clones expressed both mCherry and eGFP (Fig. 1f). These results demonstrate that pDR vector can be used to score the bidirectional transcription ability from any given DNA element. Upon comparing the transcript levels of the gene pairs chosen for the dual reporter assay in two different datasets (H1 ES cells and CD+ T cells from ENCODE) it was found that for certain pairs, the signals in the dual reporter assays did not match the bias in transcription found at the endogenous loci such as that in case of bidirectional gene pairs BDP S5, S7 and S1. Analysis of RNA-Seq data revealed that BDP S5 and BDP S7 exhibited difference in the level of transcription from the two promoters, biased towards the gene that is oriented towards mCherry in our clones. The BDP SP1 exhibited two-fold difference in expression level biased from the promoter in the sense orientation to GFP in the clone tested. However, none of these pairs exhibited a biased expression towards either of the reporter in the dual reporter assay. BDP S1 (Fig. 1) and its antisense clone in pDR BDP AS1 (Additional file 1: Figure S1C) do not show any appreciable bias towards either mCherry or GFP when tested in the pDR vector system.

**Table 1:** List of bidirectional promoters tested using the pDR vector

Gene Pair	Cloned region (hg19)	Length (bp)	Clone name
FOXM1-RHNO1	chr12:2986293–2,986,670	380	BDP-S1/AS1
LOC105369155-TMEM208	chr16:67260620–67,261,120	503	BDP-S2/AS2
TP53-WARP53	chr17:7590787–7,591,798	1000	BDP-S3/AS3
TMEM234-EIF3I	chr1:32687713–32,688,023	310	BDP-S4/AS4
CIB1-NGRN	chr15:90777156–90,777,587	431	BDP-S5/AS5
HNRNPA2B1-CBX3	chr7:26240385–26,241,390	1005	BDP-S6/AS6
CBX5-HNRNPA1	chr12:54653296–54,654,074	778	BDP-S7/AS7
GFM2-NSA2	chr5:74062655–74,063,004	350	BDP-S8/AS8

The IDs of genes within the bidirectional pair and their chromosomal locations as well as the length of intergenic distance (= bidirectional promoter) are listed. The clones in sense to eGFP were named as S1-S8 and the same clone when in sense to mCherry was named AS1-AS8 (Fig. 1, Additional file 1: Figure S1)

For BDPs S2, S3, S4, S6, S8, analysis of the RNA-Seq data revealed a bias at the endogenous loci that was skewed in favor of transcription from the promoter oriented towards GFP in the clones tested. This bias resulted in transcription that differed by at least one order of magnitude between the two promoters. In such cases there is a skew towards the GFP signal. We surmise that these sequences are inherently skewed by a margin that is extremely large and this is evident in the bias observed in the flow cytometry data. Nevertheless, in case of promoters where the bias is lower or negligible the intergenic region exhibits unbiased expression of both reporters.

### Bidirectional promoters possess distinct histone occupancy profile as compared to unidirectional promoters

**Fig. 2:**
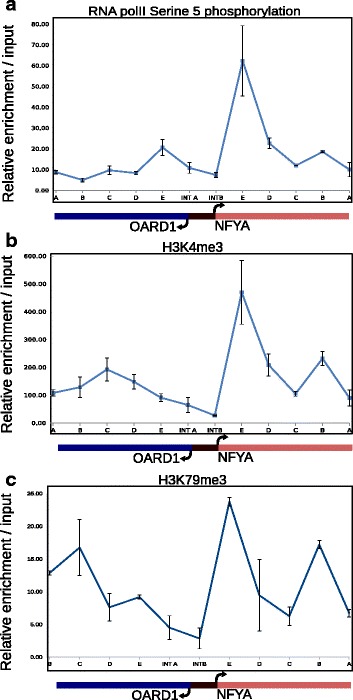
Bidirectional promoter possesses distinct epigenetic histone marks. Figure depicts ChIP analysis of the genomic locus harboring the bidirectional gene pair NFYA/OARD1 which includes 1 Kb upstream and downstream regions from the TSS of both genes. Occupancy profile of NFYA and OARD1 in Jurkat cells for Histone modifications associated with transcription activation (H3K4me3) and transcription elongation (H3K79me3) was deduced along with RNA polII phosphorylated at serine 5 using ChIP assay as described in ‘Methods’. INTA and INTB denote the two ends of the intergenic region (TSS of OARD1 and NFYA respectively), A - E indicate the location of the ChIP-qPCR primers used to map the histone modifications

Recently, polyadenylation sites found in the antisense direction to the unidirectional promoters have been shown to induce the decay of transcripts upstream to the promoter [18, 23]. Since only bidirectional promoters exhibit productive transcription in both orientations, we were interested in understanding the mechanistic differences between bidirectional and unidirectional promoters. To understand if the epigenetic mechanisms played any role in regulating bidirectional promoters we analyzed few key histone modifications at one of the bidirectional promoters. The bidirectional promoter for the gene pair NFYA/OARD1 that exhibited bidirectional transcription in the pDR vector assay (Additional file 2: Figure S2 B, C) was chosen for chromatin immunoprecipitation (ChIP) analysis in Jurkat T cells for monitoring the occupancy of the histone modifications H3K4me3, H3K79me3 and RNA pol II C-terminal domain (CTD) phosphorylated at serine 5. Expression of both these genes was confirmed by quantitative RT-PCR prior to the ChIP experiments and was found to be comparable (Additional file 2: Figure S2A). We analyzed the 1 Kb region upstream and downstream of TSS of NFYA and OARD1 gene. This region includes the common bidirectional promoter and 1 Kb of the gene body for each gene. ChIP-qPCRs were performed and data was plotted as relative occupancy normalized to input. In contrast to known enrichment pattern of these histone modifications [20], tested bidirectional promoter revealed unique enrichment profile for active promoter and elongation marks. The occupancy of RNA polII phosphorylated at serine 5 was present in either orientation flanking the intergenic region, implying that both genes were transcribed from the same element between them (Fig. 2a). The histone mark associated with active promoters viz. H3K4me3, was enriched in the both sense and antisense orientations for the bidirectional promoter (Fig. 2b). We also observed spread of the transcription elongation mark H3K79me3 in the antisense orientation (Fig. 2c). Strikingly, the profile of the epigenetic marks mirrored the levels of RNA polII implying a link between level of transcription and histone modifications at or around the bidirectional promoter.

### Genome-wide profiling of transcription associated histone modifications in bidirectional pairs

The bidirectional gene pair NFYA and OARD1 was assessed for histone modifications exhibited enrichment of histone marks that are in agreement with the transcription status of each gene within the pair. Thus, chromatin modifications appear as strong candidate for transcription regulation of bidirectional promoters. To demonstrate this further, we analyzed bidirectional gene pairs that were transcribed in an asymmetric fashion (one gene among the pair is transcribed at a much higher level than the other) as opposed to those whose expression profiles were symmetrical (both genes in a pair are either transcribed or silent) in two different cell types. To assess whether the correlation between transcription status and promoter associated histone modifications, namely H3K4me3 and H3K27ac, persist on a genome-wide scale when considering multiple bidirectional gene pairs, we analyzed 974 bidirectional gene pairs in two different cell types, namely the H1 human embryonic stem cells (H1 ES) and CD4 positive naive T cells isolated from peripheral blood (CD4^+^ T cells). The bidirectional gene pairs chosen for this analysis were previously reported [20] and have been experimentally verified to be transcribed by RNA pol**II** [20]. Wang et al. had considered ~ 1200 bidirectional gene pairs [20]. However, some of the transcripts annotated in their report are no longer maintained as bonafide transcripts in the hg19 version of ENSEMBL. A few of the bidirectional genes have been re-annotated and as a result, the locations of the genes now exist further than 1 Kb apart from its partner. After eliminating the currently un-annotated transcripts and filtering genes that are separated by more than 1000 bases we narrowed down to a list of 1070 bidirectional gene pairs that had divergent transcripts that were separated by less than 1000 bp. Since this analysis focused on divergent non-overlapping bidirectional genes, we also eliminated 95 gene pairs that showed overlapping transcription start sites (TSS) even for a single isoform. Such genes could be regulated by two independent promoters that lie in the gene body of each of the gene pairs and hence such genes were not considered for the subsequent analyses. Thus, a total of 974 gene pairs were considered for the subsequent analysis. The gene pairs were converted into transcript pairs since some of the genes had more than one isoform. In such cases, only the transcript variants that could be included in the definition of bidirectional gene pairs (transcripts originating within 1000 bp of each other arranged in a head to head orientation) were considered. In cases where more than one such combination existed, both pairs were considered independently of each other, based on transcriptional status. Further analyses or RNA-Seq and ChIP-Seq datasets was performed by considering the bidirectional transcripts that fulfill all of the criteria outlined above.

### Significant differential enrichment of histone modifications correlating with expression of individual genes from bidirectional promoters

We performed an unpaired t test to measure whether any significant differences existed between histone modification enrichments at bidirectional promoters where the genes were expressed in an asymmetric or symmetric fashion. Towards this, we further classified the genes transcribed from bidirectional promoters using their expression status. Genes that exhibited a significant difference (more than 2-fold) in the expression levels within a bidirectional gene pair were classified as asymmetrically expressed genes. Gene pairs wherein both genes were not expressed to detectable levels (Transcript per million < 1) were considered to be silent but were classified as symmetrically expressed genes. This classification was performed independently for each cell type. Thus, the genes that were asymmetric in H1ES cells can be re-classified as being symmetric in CD4 T cells or vice versa.

**Fig. 3:**
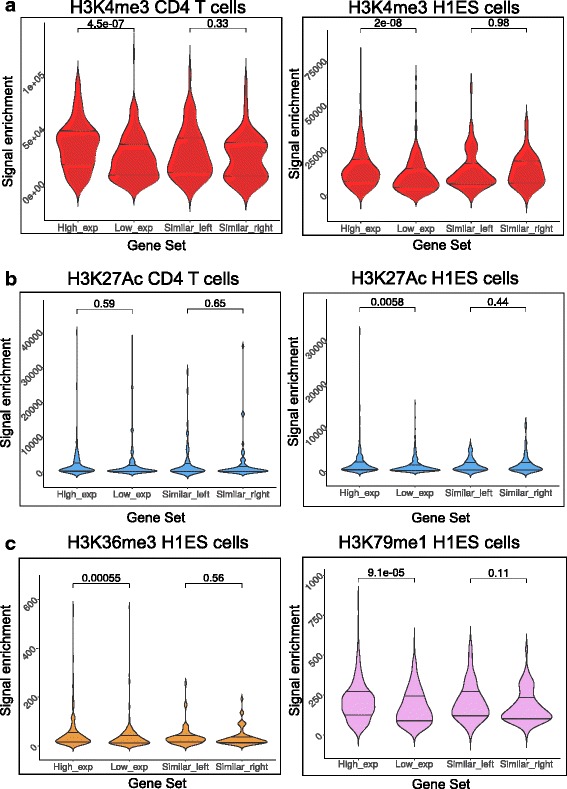
Statistical analysis of histone enrichment on bidirectional promoters classified based on transcriptional status. The figure depicts violin plots for the distributions of enrichment of various histone modifications at bidirectional promoters. The labels High_exp, Low_exp indicate gene gests that are part of an asymmetrically expressing pair with High_exp being the partner gene with high expression and Low_exp being the partner with low expression. The labels Similar_left and Similar_right indicate genes from a symmetrically expressing bidirectional pair where left and right indicate direction of transcription relative to the midpoint of the intergenic region respectively. H4K4me3 for CD4^+^T cells (*n* = 187 gene pairs for asymmetric, *n* = 58 gene pairs for symmetric) and H1 ES cells (*n* = 319 gene pairs for asymmetric, *n* = 65 gene pairs for symmetric) (**a**), H3K27Ac occupancy in CD4^+^ T cells and H1 ES cells (**b**) was analyzed. The elongation associated marks H3K436me3 (panel **c** Left) and H3K79me1 (panel **c** Right) were analyzed in H1 ES cells. Enrichment of transcription initiation mark H3K4me3 exhibits significant difference in asymmetric gene pairs in both cell types. For H1 ES cells occupancies of all histone modifications tested reveal different profiles in asymmetric and symmetric genes

The method to calculate cumulative enrichment is detailed in the methods section. For histone modifications associated with transcription initiation, the violin plots depicting the enrichment in four gene sets across two different cell types are depicted in Fig. 3 a, b, c. Each panel consists of a violin plot for a gene set that is defined as follows: 1.Genes belonging to an asymmetric gene pair and are highly transcribed as compared to their partners; 2. Genes belonging to an asymmetric gene pair that are transcribed very less as compared to their bidirectional partner; 3. Genes belonging to a symmetrically expressed bidirectional gene pair and are upstream of the midpoint of the intergenic region between the TSS; 4. Genes that are part of a symmetric gene pair but are downstream of the midpoint between the TSS. Gene pairs exhibiting lack of expression of both genes were excluded from this analysis. The histone mark H3K4me3 is associated with active promoters and was highly skewed in case of bidirectional genes with asymmetric transcription (Fig. 3a) towards the direction of the highly transcribed partner in both CD4+ T cells and H1 ES cells. Surprisingly, occupancy of H3K27Ac which is another mark associated with transcription initiation exhibited such distinct difference only in H1 ES cells and not in CD4^+^ T cells (Fig. 3b). It is noteworthy that symmetrically expressed genes exhibit virtually no differences in cumulative enrichment of transcription initiation marks across these two cell types. This implies that the histone mark H3K4me3 follows the transcriptional status of each individual gene in a bidirectional gene pair across cell types whereas H3K27Ac seems to correlate less with transcription in CD4+ cells. In a manner similar to H3K4me3, the marks associated with transcription elongation H3K79me1 and H3K36me3 also exhibit remarkable difference between asymmetrically expressed genes from a bidirectional gene pair (Fig. 3c). The symmetrically expressed genes do not show any significant difference between them implying that histone modifications closely mirrored the transcriptional status of bidirectional gene pairs. To further analyze this, we classified the bidirectional gene pairs on the basis of length of the intergenic region to precisely localize the histone modifications relative to the TSS of each gene in the pair.

### TSSs for majority of bidirectional gene pairs harbor adjacent nucleosomes at the respective + 1 positions

Since the canonical definition of bidirectional genes includes genes that are separated by less than 1000 bp [2, 24], we classified the candidate gene pairs into bins based on intergenic distance. Since we analyzed histone modifications, we classified the intergenic distance in terms of the number of nucleosomes that could be accommodated between the two TSS. In the case of bidirectional genes, the two TSS are separated by a range of genomic distances up to 1000 bp. In the case of larger intergenic distances (closer to 1000 bp), if the bidirectional gene pairs were to be two independent promoters, then the entire intergenic region is unlikely to be nucleosome free.

To account for possible presence of nucleosomes we binned the intergenic distances based on the number of nucleosomes that could be accommodated in principle. At the same time, for genes that are extremely close to each other, this gives us a measure of how close the two + 1 nucleosomes for each of the genes would be. Accordingly, the transcript pairs were binned based on integral multiples of 170 considering ~ 150 bp of DNA that can wrap around the octamer plus 20 bp as the linker region between adjacent nucleosomes. Out of 974 pairs considered, 453 transcript pairs were separated by a distance that is less than 170 bp. There is clear evidence that in the case of bidirectional genes, a single common sequence element that lies between the two TSS (in the case of non-overlapping divergent genes) regulates expression from either TSS. On the other hand, the dual reporter system pDR (Fig. 1) clearly demonstrates lack of any inherent bias towards transcription in a certain orientation when testing the bidirectional promoters. Thus, considering these two lines of evidence, it is plausible that histone modifications at or near bidirectional promoters would play a significant role in regulation of divergent transcription from such gene pairs. We hypothesized that histone modifications should mirror the direction of transcription from each bidirectional promoter depending on the relative levels of transcriptions of both genes in the pair. Since RNA polII can transcribe in both directions from a promoter to yield complete transcription in one orientation and abortive transcripts in the reverse orientation [9, 11, 12], the analysis of relative enrichment of histone modifications on each component of a bidirectional gene pair becomes relevant. It was also evident that majority of the bidirectional gene pairs lie extremely close to each other [25], therefore the tendency of such genes to be co-expressed would be especially high. Thus, analyzing each category of bidirectional genes based on the distance separating them would lead to investigating the possibility of co-regulation and its correlation with intergenic distance. Table 2 provides the number of gene pairs that are separated by distances of 1–7 nucleosomes.

**Table 2:** Numbers of bidirectional gene pairs that are separated by a specific intergenic distance

Intergenic distance	Number of bidirectional pairs
1 nucleosome (1–170 bp)	453
2 nucleosomes (170–340 bp)	245
3 nucleosomes (340–510 bp)	120
4 nucleosomes (510–680 bp)	78
5 nucleosomes (680–850 bp)	44
6 nucleosomes (850 - 1000 bp)	34
Total	974

The intergenic distance is calculated relative to the number of nucleosomes that can be accommodated between the two TSS

### Active transcription associated histone modifications are enriched on bidirectional promoters in transcription status-dependent manner

**Fig. 4:**
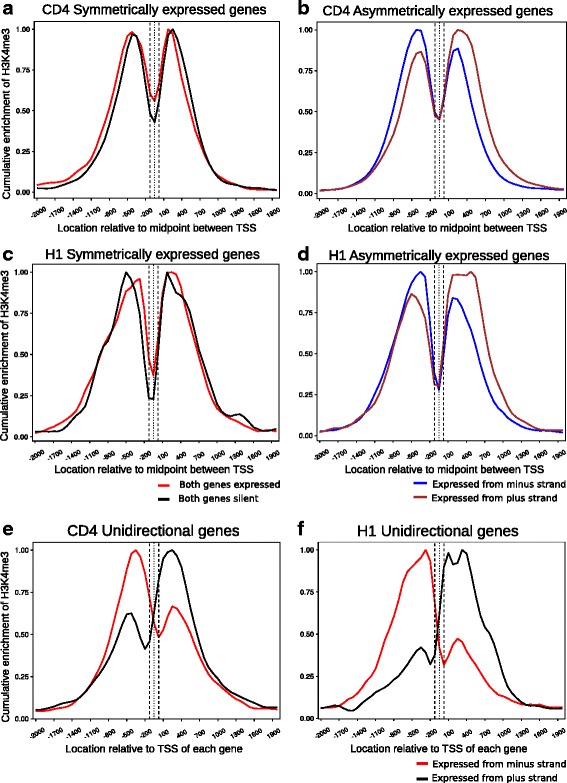
H3K4me3 marks at bidirectional genes mirror transcriptional status of gene pair. Cumulative enrichment of H3K4me3 at all bidirectional gene pairs separated by 1 nucleosome distance is shown. The gene pairs are classified into symmetrically or asymmetrically expressed gene pairs based on transcript abundances of each gene in the pair relative to its partner as described in ‘Methods’. Cumulative expression is calculated by summation of fold enrichment signal at every location in a 4 Kb window for each category and dividing by the highest value of signal in the respective category as described in ‘Methods’. ChIP-seq data from two different cell lines was analyzed, namely CD4^+^ T cells (**a**, *n* = 117; **b**, *n* = 319) and H1 human embryonic stem cells (**c**, *n* = 189; **d**, *n* = 264). Unidirectional genes are used as a control where the number of unidirectional genes is equal to the number of bidirectional gene pairs plotted. The expression range of uni-directional genes chosen is identical to the bidirectional dataset. (**e**, *n* = 500) Cumulative enrichment of H3K4me3 on unidirectional genes in CD4^+^ T cells. (**f**, *n* = 500) Cumulative enrichment of H3K4me3 on unidirectional genes in H1 ES cells. The genes transcribed on the plus and minus strands are denoted by the blue and brown colored lines respectively for the asymmetrically expressed genes. For the symmetrically expressed genes, genes that are silent are represented as the black line whereas the genes expressed from both strands are depicted with the red line. For unidirectional genes, the black and red lines represent the genes transcribed from the plus and minus strand respectively

**Fig. 5:**
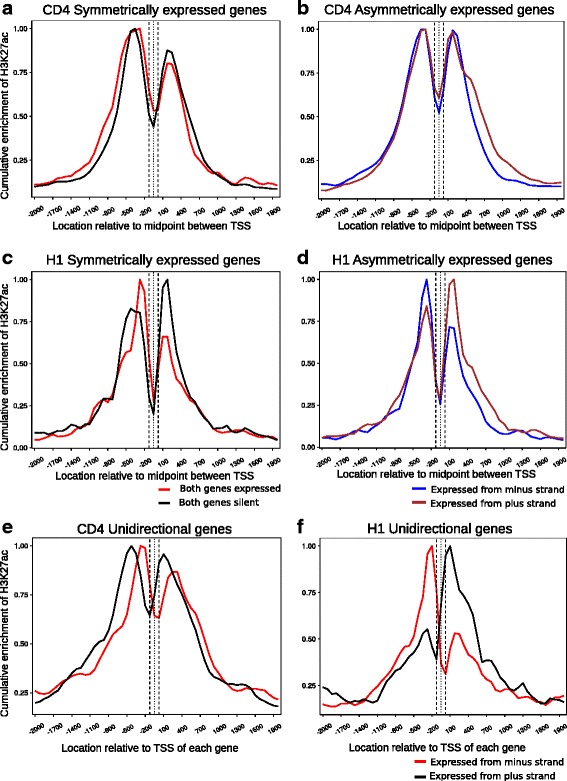
Histone H3 lysine 27 acetylation mirrors transcriptional status of bidirectional genes in H1 ES cells. Cumulative enrichment of H3K27ac at all bidirectional gene pairs separated by 1 nucleosome distance is shown. The gene pairs are classified into symmetrically or asymmetrically expressed gene pairs based on transcript abundances of each gene in the pair relative to its partner as described in ‘Methods’. Cumulative expression is calculated by summation of fold enrichment signal at every location in a 4 Kb window for each category and dividing by the highest value of signal in the respective category as described in ‘Methods’. ChIP-seq data from two different cell lines was analyzed, namely CD4^+^ T cells (**a**, *n* = 117; **b**, *n* = 319) and H1 human embryonic stem cells (**c**, *n* = 189; **d**, *n* = 264). Unidirectional genes are used as a control where the number of unidirectional genes is equal to the number of bidirectional gene pairs plotted. The expression range of unidirectional genes chosen is identical to the bidirectional dataset. (**e**, *n* =500) Cumulative enrichment of H3K27ac on unidirectional genes CD4 T cells unidirectional. (**f**, *n* = 500) Cumulative enrichment of H3K27ac on unidirectional genes in H1 ES cells. The genes transcribed on the plus and minus strands are denoted by the blue and brown colored lines respectively for the asymmetrically expressed genes. For the symmetrically expressed genes, genes that are silent are represented as the black line whereas the genes expressed from both strands are depicted with the red line. For unidirectional genes, the black and red lines represent the genes transcribed from the plus and minus strand respectively

It was evident that enrichment of H3K4me3, which is a mark associated with actively transcribing promoters, correlates extremely well with the transcriptional status of genes within a bidirectional gene pair. Cumulative enrichment of H3K4me3 calculated in a 4 Kb window that flanks the intergenic region between genes separated by a distance less than 170 bp is portrayed in Fig. 4. It is apparent that H3K4me3 enrichment is clearly correlated with the direction of transcription from asymmetrically expressed gene pairs. This is observed in both cell types analyzed here (Fig. 4 b, d). The enrichment of H3K4me3 corresponds to the gene that is being transcribed and concomitant with the direction of transcription. For genes that are transcribed on the plus strand, H3K4me3 peak is observed just upstream of the TSS and the pattern is mirrored for genes transcribed from the minus strand. However, for symmetrically transcribed genes, the H3K4me3 enrichment is completely different. Whereas the H3K4me3 profile for asymmetric genes and unidirectional genes is identical (Fig. 4 b, d, e, f), symmetrically transcribed genes exhibit a bimodal enrichment (Fig. 4 a, c). Since this is cumulative enrichment, it is independent of the absolute amount of a particular histone modification on each gene pair. Thus, even gene pairs wherein both partners are silent exhibit baseline level of H3K4me3 enrichment. It is of interest to note that such a profile is also similar to symmetrically transcribed genes rather than unidirectional genes. This correlation seems to hold true for genes that are separated by a distance larger than 170 bp as well, (Additional file 3: Figure S3, Additional file 4: Figure S4) and is consistent for a distinct profile for asymmetrically expressed and symmetrically expressed genes in both cell types. As expected from our statistical analysis, enrichment of H3K27ac, another epigenetic mark associated with active transcription, varies significantly between the two cell types analyzed (Fig. 5 a-d). H3K27ac enrichment follows transcription status in H1 ES for asymmetric gene pairs (Fig. 5 c, d). In CD4^+^ T cells, asymmetric gene pairs exhibit the same profile of H3K27ac occupancy as that of symmetric gene pairs (Fig. 5 a, b). However, it is interesting to note that even for unidirectional genes in CD4^+^ T cells (Fig. 5e), H3K27ac occupancy profile is reminiscent of symmetrically expressed bidirectional genes (Fig. 5a) implying that H3K27ac profiles may fluctuate depending upon the cell type. As the unidirectional genes here are randomly sampled and expression matched to the bidirectional gene pairs, no functional link between the two datasets is expected. Hence H3K27ac profiles in CD4^+^ T cells may be distinct from those in H1 ES cells independent of gene organization. As is evident from the H3K27ac enrichment at gene pairs that are separated by larger distances (Additional file 5: Figure S5, Additional file 6: Figure S6), the H3K27ac occupancy profile clearly mirrors the transcriptional status in case of asymmetric genes in H1 ES cells but not so clearly in CD4^+^ T cells.

### Histone modifications associated with transcription elongation are enriched on bidirectional genes in transcription status-dependent manner

**Fig. 6:**
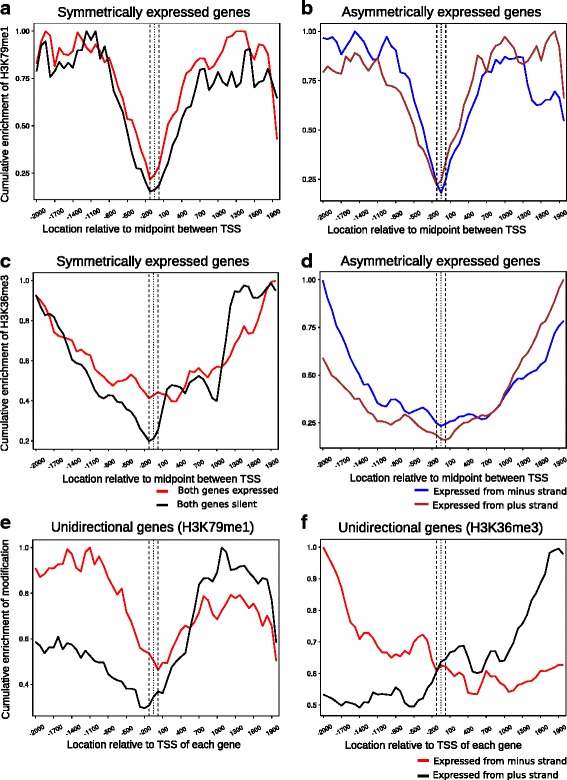
Modifications associated with transcription elongation follow the transcriptional status of bidirectional genes in H1 ES cells. Cumulative enrichment of transcription elongation associated histone marks at all bidirectional gene pairs separated by one nucleosome distance. The gene pairs are classified into symmetrically or asymmetrically expressed gene pairs based on transcript abundances of each gene in the pair relative to its partner as described in ‘ Methods’. Cumulative expression is calculated by summation of fold enrichment signal at every location in a 4 Kb window for each category and dividing by the highest value of signal in the respective category as described in ‘Methods’. ChIP-seq data for two different Histone modifications associated with transcription elongation was analyzed, namely H3K79me1 (**a**, *n* = 117; **b**, *n* = 319) and H3K36me3 (**c**, **n** = 189; **d**, *n* = 264). Unidirectional genes were used as a control such that the number of unidirectional genes is equal to the number of bidirectional gene pairs plotted. The expression range of uni-directional genes chosen is identical to the bidirectional dataset. (**e**, *n* = 500) Cumulative enrichment of H3K79me1 on unidirectional promoters. (**f**, *n* = 500) Cumulative enrichment of H3K36me3 on unidirectional promoters. The genes transcribed on the plus and minus strands are denoted by the blue and brown colored lines respectively for the asymmetrically expressed genes. For the symmetrically expressed genes, genes that are silent are represented as the black line whereas the genes expressed from both strands are depicted with the red line. For unidirectional genes, the black and red lines represent the genes transcribed from the plus and minus strand respectively

The occupancy profiles of the two elongation associated modifications namely H3K79me1 and H3K36me3 were analyzed for the same set of 974 bidirectional gene pairs in the H1 embryonic stem cell data. Parallel datasets in CD4^+^ T cells were unavailable and hence could not be compared. In case of the bidirectional gene pairs, H3K79me1 occupancy profile follows the transcriptional status of each individual gene (Fig. 6 a, b). This correlation is even more striking for H3K36me3 (Fig. 6 c, d). For symmetrically expressed gene pairs the transcription elongation associated modifications exhibit a bimodal distribution. Furthermore, these profiles are specific to symmetrically expressed bidirectional promoters and unidirectional genes do not exhibit such a profile. Occupancy profiles for the modifications H3K79me1 and H3K36me3 on unidirectional genes strongly mirrored the transcription status of the genes (Fig. 6e and f respectively). The profile of elongation associated mark H3K36me3 for asymmetrically expressed bidirectional genes is similar to known unidirectional genes with a similar expression level (Fig. 6 d, f). The difference in occupancy profiles for H3K79me1 is not as striking but appreciable nevertheless (Fig. 6 b, e). This holds true for all bidirectional gene pairs tested irrespective of the intergenic distance separating them (Additional file 7: Figure S7, Additional file 8: Figure S8). Based on all of the above occupancy profiles we conclude that histone modifications mirror the transcription status of bidirectional genes and could potentially serve as regulatory switches that determine symmetric or asymmetric transcription from such regions rather than any inherent bias in the promoter region itself towards any specific orientation of transcription. Collectively, these analyses indicate that similar to histone modifications associated with transcription activation, histone modifications that are signatures of maturing transcription also correlated with the transcriptional status of each gene in the bidirectional pairs tested.

### Histone modifications associated with transcription elongation are functionally associated with bidirectional transcription

**Fig. 7:**
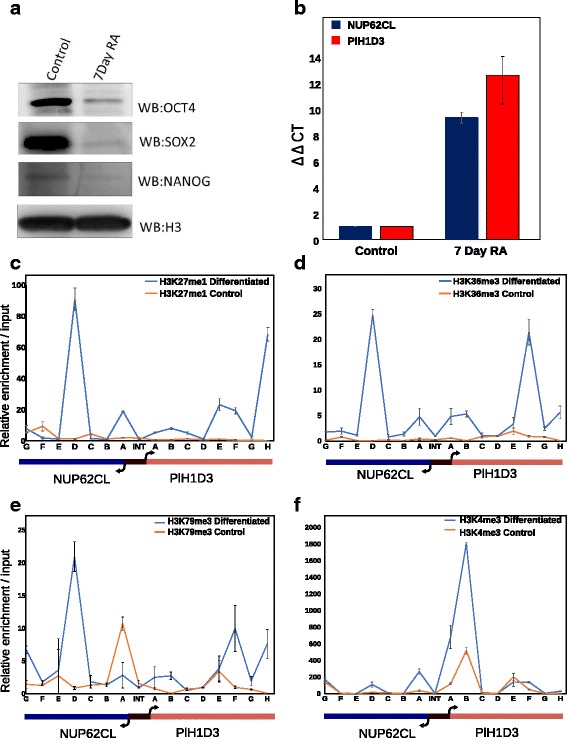
Histone modification profile of bidirectional gene pair during NT2D1 EC cell differentiation. **a** Immunoblot for Oct4, Nanog and Sox2 for undifferentiated NT2D1 cells and 7 day RA differentiated NT2D1 cells was performed as described in ‘Methods’. After 7 days all these pluripotency factors show diminished expression indicative of differentiated cells. **b** Relative expression of X linked gene pair NUP62CL and PIH1D3 in undifferentiated and 7 day differentiated NT2D1 cells, normalized to GAPDH in each sample. (**c**, **d**, **e**, **f**) Relative enrichment of transcription elongation associated histone marks H3K27me1, H3K36me3, H3K79me3 and H3K4me3 in the gene pair NUP62CL and PIH1D3 upon 7 day RA mediated differentiation in NT2D1 cells

To address whether the signature histone modifications discussed earlier would be functionally associated with bidirectional transcription in a biological context, we monitored their occurrence on a bidirectional gene pair upon retinoic acid (RA) mediated differentiation of NT2D1 human embryonic carcinoma cells. These cells behave similar to embryonic stem cells since they express all pluripotency factors and can be differentiated into specific cell types upon providing specific signals [26]. We differentiated NT2D1 cells using RA treatment for 7 days and analyzed gene expression from a few bidirectional promoters. Immunoblot analysis of pluripotency factors revealed decrease in the expression of OCT4, SOX2 and Nanog (Fig. 7a), confirming the differentiation of NT2D1 cells. We specifically selected X-linked genes for this analysis as the NT2D1 cell line contains a single copy of the X chromosome (Additional file 9: Figure S9). The pair NUP62CL-PIH1D3 exhibited maximum increase in transcription post RA mediated differentiation among all the pairs tested. Therefore this pair was selected a target for ChIP analysis of selected histone modifications. Using qRT-PCR analysis we confirmed that gene expression for the pair NUP62CL-PIH1D3 increased nearly 10-fold upon differentiation (Fig. 7b). Next, ChIP analysis for H3K79me3, H3K36me3 and H3K27me1 was performed in control and 7-day differentiated cells. A region spanning 2 Kb on either side of the TSS for each gene was analyzed for occupancy of various histone modifications which included the intergenic bidirectional promoter (INT). A concomitant increase in H3K79me3, H3K36me3 and H3K27me1 in the gene bodies of both the genes was observed (Fig. 7 c-e) which strongly correlated with the increase in transcription from these genes. As expected, the intergenic region (INT) did not show any enrichment of the transcription elongation associated marks. Interestingly, an increase in H3K4me3 occupancy was observed in the differentiated NT2D1 cells that mirrored the initiation of transcription from the gene pair, though much lower on NUP62CL as compared to PIH1D3 (Fig. 7f). Surprisingly, profile of H3K4me3 occupancy at one location proximal to the TSS of PlH1D3 exhibited H3K4me3 enrichment even in undifferentiated cells, albeit at lower level (Fig. 7f). The same location exhibits a marked increase in H3K4me3 upon gene activation and this increase is observed over the entire intergenic region (proximal to TSS of both the gene pairs tested). Thus, histone modifications seem to decorate bidirectional promoters in a manner that mirrors the transcription status of the genes.

## Discussion

The regulation of bidirectional transcription is not clearly understood and this study aims at answering this question from an epigenetic perspective. A luciferase reporter based system was typically used to characterize bidirectional promoters [1]. However, a major limitation of this system is that one can score for only unidirectional firing of the promoter at one time. To overcome this limitation, we constructed a vector system that can be used for studying bidirectional promoters wherein any DNA fragment acting as a bonafide bidirectional promoter can transcribe two reporters (GFP and mCherry) simultaneously and a unidirectional promoter can transcribe either of the two reporters based on its orientation. We have named this dual reporter construct as the pDR vector. To validate this vector in live cells we cloned the CMV promoter that is a well-characterized unidirectional promoter [27]. CMV promoter drives the expression of GFP or mCherry depending upon whether it was cloned in sense or antisense orientations in between the two reporters and similar results were obtained using an endogenous GAPDH unidirectional promoter. These results suggest that any unidirectional promoter would behave in similar manner. Further, we cloned 8 representative bidirectional promoters from human genome in pDR vector and characterized them in vivo by microscopy and flow cytometry. Thus, the dual reporter vector can be used to identify regulatory elements and the role of different transcription factors in the regulation of bidirectional promoters.

Previous studies have already established that bidirectional promoters exhibit specific enrichment of active transcription associated chromatin signatures [28]. The occupancy of H3K4me3, usually associated with active promoters in antisense direction is reported and it is thought to be required for initiation of antisense transcription from human promoters [9, 29]. However, the most striking difference between any unidirectional and bidirectional promoter is the productive transcription elongation in antisense direction (relative to any one direction of transcription), which is specific to bidirectional promoters. None of the studies till date have provided mechanistic insights into this striking feature of transcription elongation from bidirectional promoters. Although Seila et. al [12] mention that bidirectional transcription is a hallmark of many TSSs, however the histone modifications like H3K4me3 and elongation marks such as H3K36me3 are not found in bidirectional fashion. The antisense transcription from most unidirectional TSS is abortive and is not productive. Thus, the role of histone modifications contributing to chromatin architecture in the vicinity of bidirectional promoters is unclear. To test the role of histone modifications we performed ChIP analysis in Jurkat T cells for the NFYA-OARD1 bidirectional gene pair. ChIP analysis revealed that the bidirectional promoter of NFYA-OARD1 harbors a unique distribution of active transcription associated promoter and elongation marks in both orientations correlating very well with the presence of RNA polII that harbors the serine 5 phosphorylation on its C-terminal domain (CTD). This prompted us to propose that this unique distribution of active transcription marks on bidirectional promoters and their respective gene body might be responsible for transcription elongation in both orientations from such loci, leading to production of complete transcripts. The apparent discrepancy of enrichment for each IP at position ‘E’ in Fig. 2 could be explained as a local effect due to either higher accessibility of chromatin for immunoprecipitation at that particular locus, or higher amplification efficiency of the pair of oligonucleotide primers used for amplifying the specific DNA sequence. At a genome-wide scale, our study establishes the fact that for bidirectional promoters, the histone modification landscape mirrors the transcriptional status of each gene in the pair.. This is a crucial observation because a large fraction of bidirectional genes (~ 49% of promoter pairs tested in this analysis) lie in close proximity to each other (< 170 bp). Thus, what can be considered as potentially adjoining nucleosomes exhibit a strikingly different enrichment of histone modifications solely based on transcriptional status. This is especially true for asymmetrically expressed gene pairs where histone marks do not spread over to their adjacent nucleosome.

As a category of transcriptionally distinct gene sets, the symmetric and asymmetric transcription units show a distinct difference from each other. It would be difficult to extend this analysis quantitatively to each particular gene pair since quantitation of histone modifications from ChIP-Seq data is not possible at the same resolution as quantitation of transcripts from RNA-Seq. As such, individual gene pairs cannot be tested for a quantum of difference in the extent of histone modification that correlates directly to a difference in transcript abundance, as the accuracy of one estimation is lower than the other. However, if analyzed as a group then the differences between gene sets become apparent. Since the promoter element between a bidirectional gene pair has been previously reported to a single unit, whose disruption leads to loss of expression from both transcripts from the pair [1], asymmetric transcription factor binding can explain only part of the regulatory mechanism, especially for loci wherein the genes are in close proximity to each other.

A mechanism of transcription using transcription factors (TFs) as the primary driving force for transcription regulation is not sufficient to explain these stark differences between histone modification enrichment patterns. Transcription factors have specific sites that they recognize, however, the sites that have been reported to enriched on bidirectional promoters are for proteins such as SP1, YY1 etc. [30], which are generic TFs. This in itself is not surprising given the fact that many bidirectional genes have housekeeping functions and are geared for bursts of transcription [1, 2, 22]. However, the maintenance of a skewed histone modification profile which is completely dependent on transcription status has to be part of an actively maintained regulatory mechanism. Since the promoter element between any two bidirectional genes (defined in the context of this work) has been demonstrated to be a single regulatory element [1]. Thus, specific set of histone modifications seems to be one possible mechanism to maintain asymmetric expression status in a situation where all other things are equal. However, it is unclear whether these histone modification profiles are a cause or consequence of transcriptional status. The difference in behavior of the bidirectional promoters when assessed in the pDR vector as opposed to the endogenous loci could be due to a difference in histone modifications accrued by the plasmid in HEK293T cells as opposed to the endogenous locus. Nonetheless, it is abundantly clear that bidirectional transcription is an inherent property of the promoters tested. The altered ‘behavior’ of bidirectional promoters when removed from their endogenous loci underscores the role of histone modifications in maintaining transcriptional status according to the regulatory mechanisms governing cell type specific regulation from bidirectional promoters. This is applicable especially in the case of the promoters that show unbiased bidirectional transcription when cloned in the pDR vector as opposed to transcription activity analyzed at their endogenous loci.

It is equally probable that a stark difference a difference in transcription activity would result into a difference in the histone occupancy profiles at the endogenous loci in the cell.

**Fig. 8:**
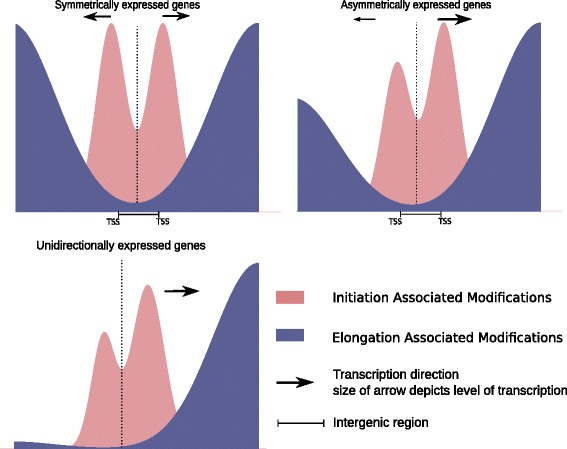
Model depicting the unique enrichment of histone modifications on bidirectional promoter. Distribution of active promoter mark and elongation marks show unique enrichment on bidirectional promoter which correlates with transcriptional status of bidirectional promoter regulated gene pairs. Genes that are symmetrically expressed from bidirectional promoter show bimodal enrichment of transcription associated histone marks. In contrast, asymmetrically expressed genes behave like unidirectional promoters with respect to direction of active mature transcription

Either way, correlation between the histone modification profile and transcriptional profile is evident. Thus, it is possible that these modifications help in preserving transcriptional status of the gene pair that overrides the inherent tendency of bidirectional promoters to transcribe in both orientations. We confirmed a functional connection between histone marks associated with elongation for at least one gene pair. We used RA mediated differentiation of the human embryonic carcinoma NT2D1 cells as model system. Based on a 10-fold differential expression for the NUP62CL-PIH1D3 upon RA mediated differentiation, we selected this gene pair for ChIP analysis of transcription elongation marks H3K79me3, H3K36me3 and H3K27me1. Such stark difference in expression for the gene pair in the two states of differentiation rendered it an attractive candidate for assessment of histone marks that are dependent on active transcription. We analyzed the enrichment of these elongation marks in 2 Kb upstream and downstream regions from TSS of NUP62CL-PIH1D3 gene pair including the intergenic region. Interestingly, all three histone marks exhibited significant enrichment downstream to TSS on both gene bodies, which explains the significance of bimodal distribution of elongation marks in productive bidirectional transcription from sense and antisense orientations. This is the first instance where a direct correlation of histone modification marks in regulating the transcriptional state of the bidirectional genes driven by the bidirectional promoters has been proposed. The results presented here identify an epigenetic histone modification signature of bidirectional promoters that sets them apart from all other transcribing loci in the genome (schematically depicted in Fig. 8). These results provide a pattern of histone modifications on bidirectional promoters that can be used to predict if two genes present in close proximity might be co-regulated through a bidirectional promoter.

Various mechanisms have been discussed and debated in the past regarding whether the histone modification state is a cause or a consequence of transcription or vice versa. However, until now this enigmatic correlation is not unequivocally answered [31]. The molecular mechanism(s) of such putative correlation of epigenetic regulation and bidirectional transcription are far from clear. Results of our dual reporter assays using GAPDH promoter and bidirectional promoters suggest that the short intergenic region is almost solely responsible for productive transcription in both orientations from the bidirectional pair. If the mere act of juxtaposing two genes in head-to-head orientation were to be enough to deposit histone marks in the gene bodies of eGFP and mCherry, two genes that do not naturally express in any mammalian system, then the role of ascribing bidirectional transcription status lies solely with the intergenic region and the bimodal distribution of histone marks presumably follows as a consequence of this. This is further supported by our observation that in a situation wherein only one out of the two genes in a pair is transcribed, the histone marks seem to mirror transcriptional status as opposed to merely the presence of a bidirectionally responsive promoter element. This opens a very interesting possibility that productive transcription in the silenced gene is suppressed by a separate mechanism that does not allow the deposition of histone marks that enhance transcription. Thus, it is possible that histone deposition can act as a memory of active transcription status. However, the precise mechanism regulating the downstream deposition of histone modification marks that mirror the transcriptional status of each gene in the bidirectional gene pair remains yet to be understood.

## Conclusions

### Chromatin modification marks mirror transcriptional status of bidirectional genes, presumably leading to maintenance of transcription status

We report that the epigenetic modifications of nucleosomes at the bidirectional promoters mirror the transcriptional status of each gene seemingly independently of its partner. This is especially observed for genes where the difference in expression between the partners is more than 2-fold. This profile of histone modifications holds true for all bidirectional gene pairs analyzed, most strikingly for gene pairs that are separated by just 1 nucleosome distance. This implies that adjacent nucleosomes possess modifications that correlate with transcriptional status of the gene. The differences in enrichment of modifications are significant independent of the intergenic distance. We therefore hypothesize that while most bidirectional promoters may not be inherently biased towards any given orientation for initiating and completing transcription, epigenetic modifications in the native chromatin context might be responsible for maintenance of symmetric or asymmetric expression profiles, in conjunction with transcription factors and associated machinery. We demonstrate the correlation between a specific histone profile and expression status by analyzing bidirectional transcription in two different cell types. In the case of undifferentiated embryonic carcinoma cells, we show a concomitant increase of a bimodal histone modification profile that only appears after transcriptional upregulation at the locus tested. Thus, we hypothesize that bidirectional transcription may be inherently unbiased towards a particular orientation but histone modifications that are dynamically controlled may play a role in maintaining symmetry/asymmetry of transcription status at bidirectional promoters.

## Additional files

Additional file 1: **Figure S1.** Design of dual reporter vector and bidirectional transcription from bidirectional promoters cloned in antisense orientation. (A) The strategy for introducing eGFP and mCherry under a common regulatory DNA element is shown. This vector construct is designed to provide a quantitative readout in live cells based on strand-specific promoter activity. Intense red and green colors indicate direction of promoter activity. Three constructs are shown in the scheme, in middle is pDR vector with no promoter element, Right side shows construct with CMV promoter antisense to mCherry, left side shows construct with CMV promoter antisense to eGFP. (B) Clones from Fig. 1 in their antisense orientation show the same outcome. (PDF 1427 kb)

Additional file 2: **Figure S2.** Characterization of the bidirectional gene pair NFYA-OARD1. (A) The figure shows relative expression of NFYA-OARD1 in Jurkat cells as measured by quantitative RT-PCR analysis. The expression levels are normalized to GAPDH (B) Fluorescence images of the NFYA-OARD1 intergenic region when cloned into the pDR vector. The orientation of each clone with respect to NFYA is depicted below each set of images. Scale bar denotes 400 μm. (C) Flow cytometry plots of cells transfected with NFYA-OARD1 cloned into pDR vector in which NFYA is in sense orientation to mCherry. As a control NFYA cloned into eGFP-N1 vector was used wherein NFYA drives the expression of GFP. The axes denoting mCherry and eGFP are depicted adjacent to the plots. (PDF 1434 kb)

Additional file 3: **Figure S3.** H3K4me3 distribution on bidirectional gene with different intergenic distances in H1 ES cells. The figure shows enrichment of H3K4me3 at the bidirectional genes pairs with intergenic distance upto 1000 bp. Intergenic distance is represented as the number of nucleosomes that could potentially be accommodated. Data are shown for the gene pairs which have intergenic region that could contain 2 to 6 nucleosomes assuming 170 bp length for wrapping around each octamer and inclusive of the 20 bp linker. Cumulative expression is calculated by summation of fold enrichment signal at every location in a 4 KB window for each category and dividing by the highest value of signal in the respective category as described in ‘Methods’. (A) Cumulative enrichment of H3K4me3 on bidirectional genes which are asymmetric with respect to their expression profiles. (B) Cumulative enrichment of H3K4me3 on bidirectional genes whose expression profiles are symmetric. (PDF 347 kb)

Additional file 4: **Figure S4.** H3K4me3 distribution on bidirectional gene with different intergenic region in CD4^+^ T cells. The figure shows enrichment of H3K4me3 at the bidirectional genes pairs with intergenic distance up to 1000 bp. Intergenic distance is represented as the number of nucleosomes that could potentially be accommodated. Data are shown for the gene pairs which have intergenic region that could contain 2 to 6 nucleosomes assuming 170 bp length for wrapping around each octamer and inclusive of the 20 bp linker. Cumulative expression is calculated by summation of fold enrichment signal at every location in a 4 Kb window for each category and dividing by the highest value of signal in the respective category as described in ‘Methods’. (A) Cumulative enrichment of H3K4me3 on bidirectional genes which are asymmetric with respect to their expression. (B) Cumulative enrichment of H3K4me3 on bidirectional genes which are symmetric with respect to their expression. (PDF 1048 kb)

Additional file 5: **Figure S5.** H3K27ac distribution on bidirectional gene with different intergenic region in H1 ES cells. The figure shows enrichment of H3K27ac at the bidirectional genes pairs with intergenic distance up to 1000 bp. Intergenic distance is represented as the number of nucleosomes that could potentially be accommodated. Data are shown for the gene pairs which have intergenic region that could contain 2 to 6 nucleosomes assuming 170 bp length for wrapping around each octamer and inclusive of the 20 bp linker. Cumulative expression is calculated by summation of fold enrichment signal at every location in a 4 Kb window for each category and dividing by the highest value of signal in the respective category as described in ‘Methods’. (A) Cumulative enrichment of H3K27ac on bidirectional genes which are asymmetric with respect to their expression profiles. (B) Cumulative enrichment of H3K27ac on bidirectional genes which are symmetric with respect to their expression profiles. (PDF 1135 kb)

Additional file 6: **Figure S6.** H3K27ac distribution on bidirectional gene with different intergenic region in CD4 T cells. The figure shows enrichment of H3K27ac at the bidirectional genes pairs with intergenic distance up to 1000 bp. Intergenic distance is represented as the number of nucleosomes that could potentially be accommodated. Data are shown for the gene pairs which have intergenic region that could contain 2 to 6 nucleosomes assuming 170 bp length for wrapping around each octamer and inclusive of 20 bp linker. Cumulative expression is calculated by summation of fold enrichment signal at every location in a 4 Kb window for each category and dividing by the highest value of signal in the respective category as described in ‘Methods’. (A) Cumulative enrichment of H3K27ac on bidirectional genes which are asymmetric with respect to their expression profiles. (B) Cumulative enrichment of H3K27ac on bidirectional genes which are symmetric with respect to their expression profiles. (PDF 819 kb)

Additional file 7: **Figure S7.** H3K79me1 distribution on bidirectional gene with different intergenic region in H1 ES cells. The figure shows enrichment of H3K79me1 at the bidirectional genes pairs with intergenic distance up to 1000 bp. Intergenic distance is represented as the number of nucleosomes that could potentially be accommodated. Data are shown for the gene pairs which have intergenic region that could contain 2 to 6 nucleosomes assuming 170 bp length for wrapping around each octamer and inclusive of 20 bp linker. Cumulative expression is calculated by summation of fold enrichment signal at every location in a 4 KB window for each category and dividing by the highest value of signal in the respective category as described in ‘Methods’. (A) Cumulative enrichment of H3K79me1 on bidirectional genes which are asymmetric with respect to their expression profiles. (B) Cumulative enrichment of H3K79me1 on bidirectional genes which are symmetric with respect to their expression profiles. (PDF 831 kb)

Additional file 8: **Figure S8.** H3K36me3 distribution on bidirectional gene with different intergenic region in H1 ES cells. The figure shows enrichment of H3K79me1 at the bidirectional genes pairs with intergenic distance up to 1000 bp. Intergenic distance is represented as the number of nucleosomes that could potentially be accommodated. Data are shown for the gene pairs which have intergenic region that could contain 2 to 6 nucleosomes assuming 170 bp length for wrapping around each octamer and inclusive of 20 bp linker. Cumulative expression is calculated by summation of fold enrichment signal at every location in a 4 KB window for each category and dividing by the highest value of signal in the respective category as described in ‘Methods’. (A) Cumulative enrichment of H3K36me3 on bidirectional genes which are asymmetric with respect to their expression profiles. (B) Cumulative enrichment of H3K36me3 on bidirectional genes which are symmetric with respect to their expression profiles. (PDF 829 kb)

Additional file 9: Expression profile 10 bidirectional gene pairs upon RA mediated differentiation of NT2D-1 cells. These gene pairs were tested for expression differences with real-time PCR upon RA mediated differentiation as described in Methods. (PDF 58 kb)

## Abbreviations

BDP-AS1: Bidirectional promoter clone 1 in antisense orientation
BDP-S1: Bidirectional promoter clone 1 in sense orientation
CTD: C-terminal domain
INT: Intergenic region between bidirectional gene pair
pDR: Plasmid with dual reporters (eGFP and mCherry)
polII: polymerase II
TF: Transcription Factor
TPM: Transcripts per Million
TSS: Transcription start site
