# Supplementary figures and images for: Bidirectional promoters exhibit characteristic chromatin modification signature associated with transcription elongation in both sense and antisense directions
Source: BMC Genomics. 2018 May 2;19:313. doi: 10.1186/s12864-018-4697-7 (PMC5930751; doi:10.1186/s12864-018-4697-7)

# Figure S1

## A

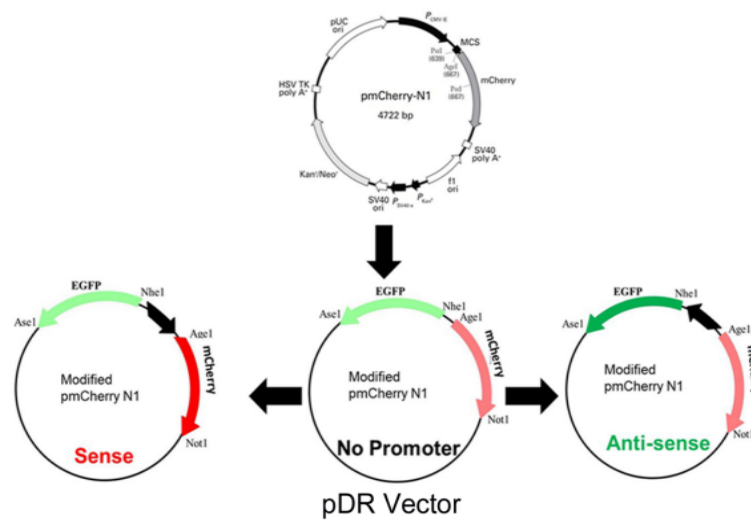

## B

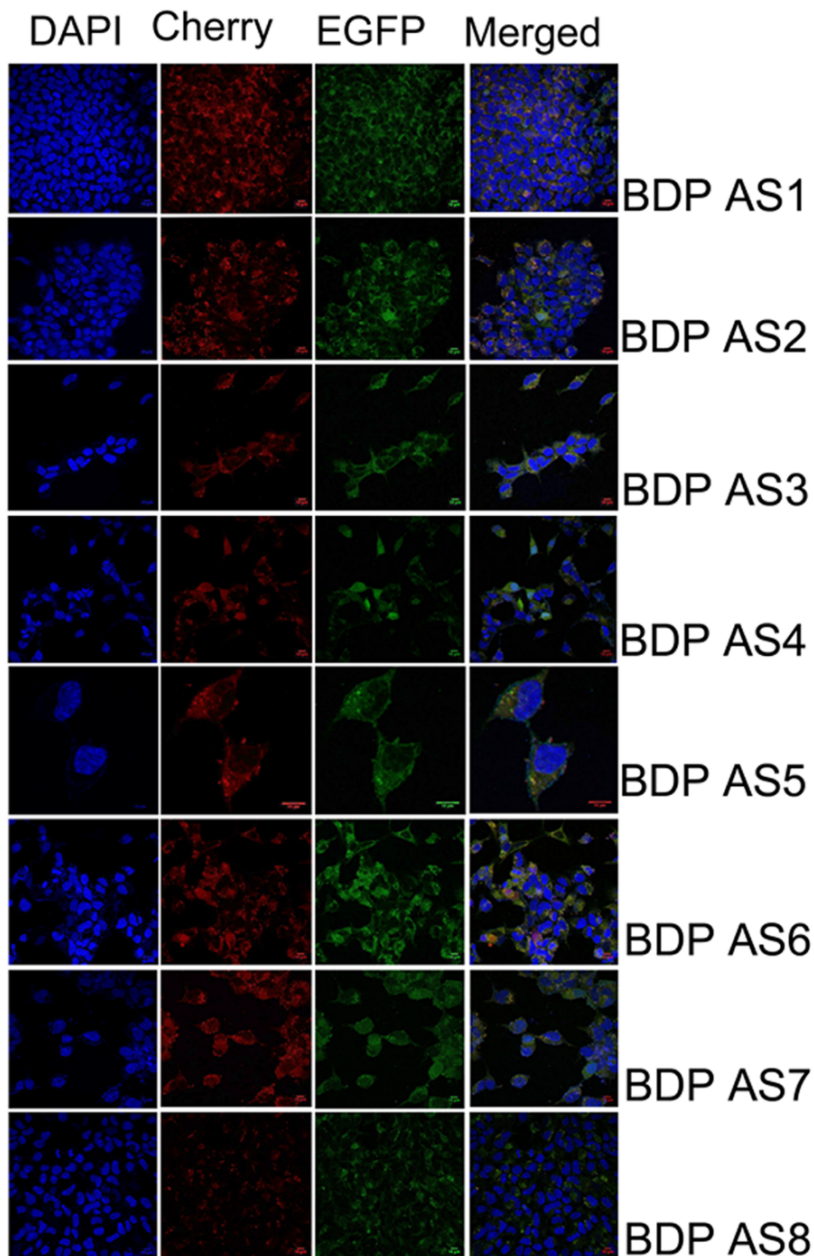

## C

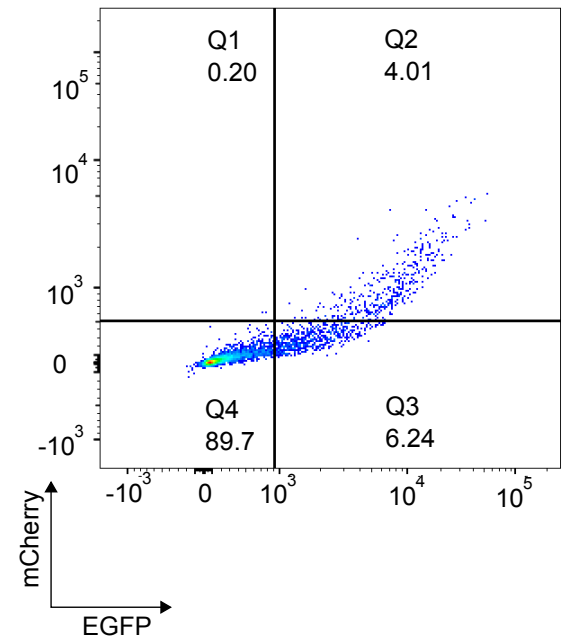

BDP AS1  
Flow Cytometry

Supplement: Supplementary file 1 — Figure S1. Design of dual reporter vector and bidirectional transcription from bidirectional promoters cloned in antisense orientation. (A) The strategy for introducing eGFP and mCherry under a common regulatory DNA element is shown. This vector construct is designed to provide a quantitative readout in live cells based on strand-specific promoter activity. Intense red and green colors indicate direction of promoter activity. Three constructs are shown in the scheme, in middle is pDR vector with no promoter element, Right side shows construct with CMV promoter antisense to mCherry, left side shows construct with CMV promoter antisense to eGFP. (B) Clones from Fig. 1 in their antisense orientation show the same outcome. (PDF 1427 kb) [file 12864_2018_4697_MOESM1_ESM.pdf]

# Figure S2

A

Relative expression of NFYA and OARD1  
in Jurkat cells

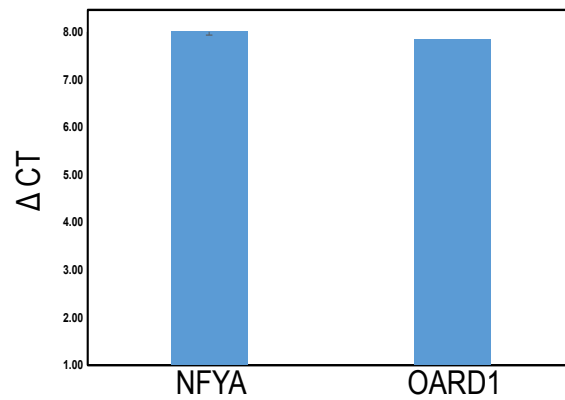

B

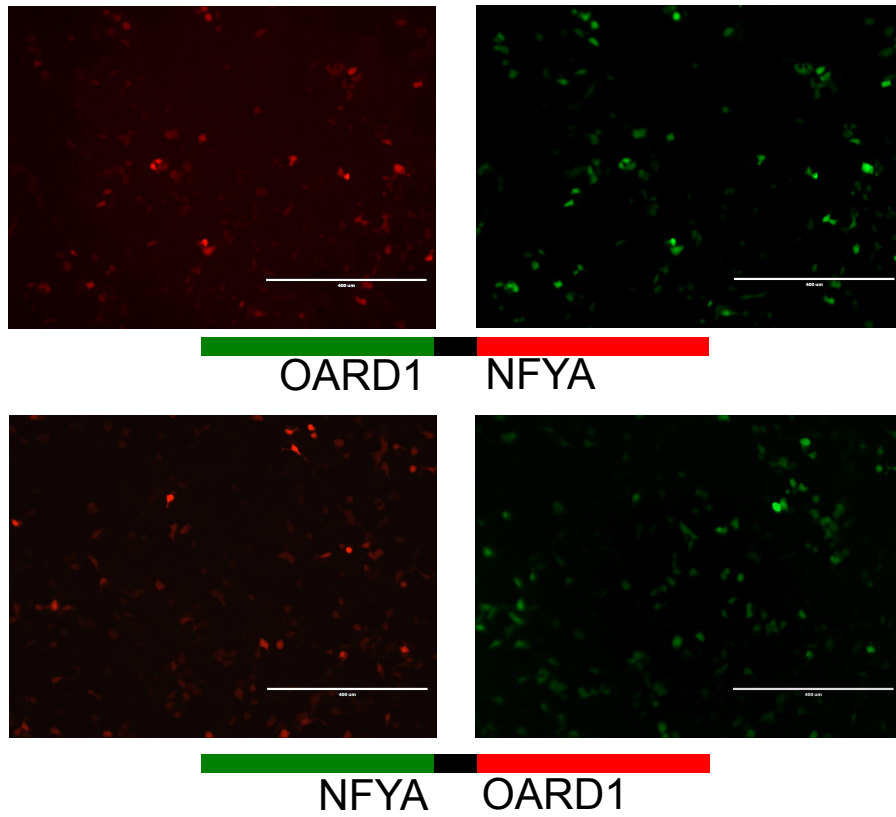

C

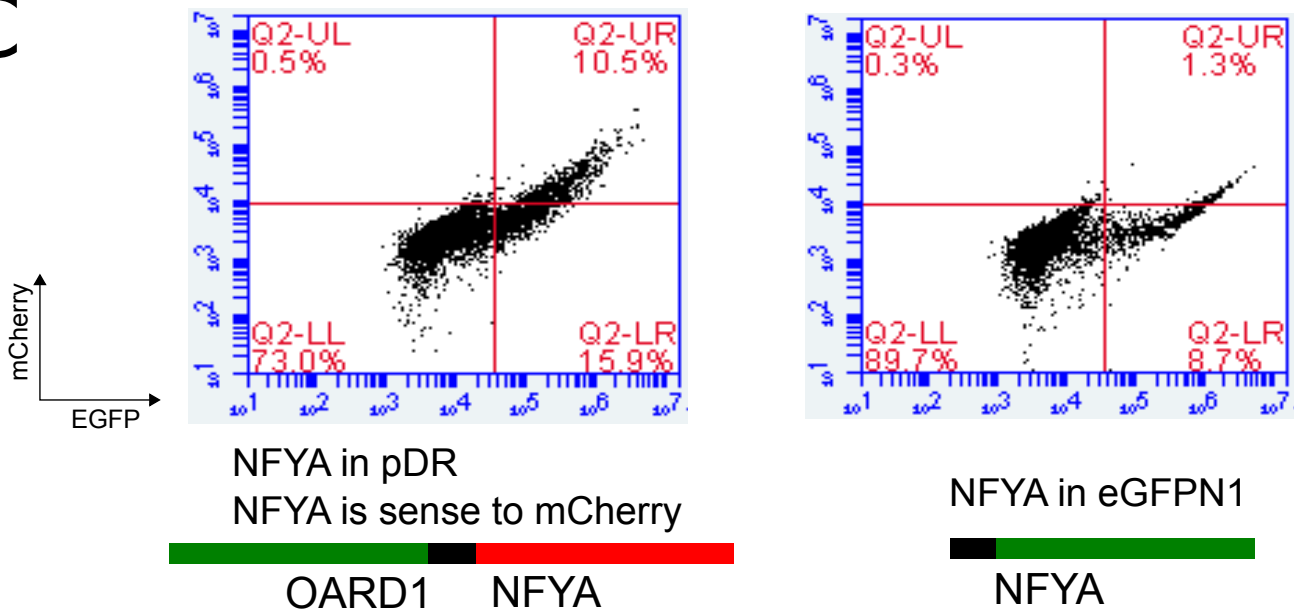

Supplement: Supplementary file 2 — Figure S2. Characterization of the bidirectional gene pair NFYA-OARD1. (A) The figure shows relative expression of NFYA-OARD1 in Jurkat cells as measured by quantitative RT-PCR analysis. The expression levels are normalized to GAPDH (B) Fluorescence images of the NFYA-OARD1 intergenic region when cloned into the pDR vector. The orientation of each clone with respect to NFYA is depicted below each set of images. Scale bar denotes 400 μm. (C) Flow cytometry plots of cells transfected with NFYA-OARD1 cloned into pDR vector in which NFYA is in sense orientation to mCherry. As a control NFYA cloned into eGFP-N1 vector was used wherein NFYA drives the expression of GFP. The axes denoting mCherry and eGFP are depicted adjacent to the plots. (PDF 1434 kb) [file 12864_2018_4697_MOESM2_ESM.pdf]

# Figure S3

## A

### Asymmetrically expressed genes

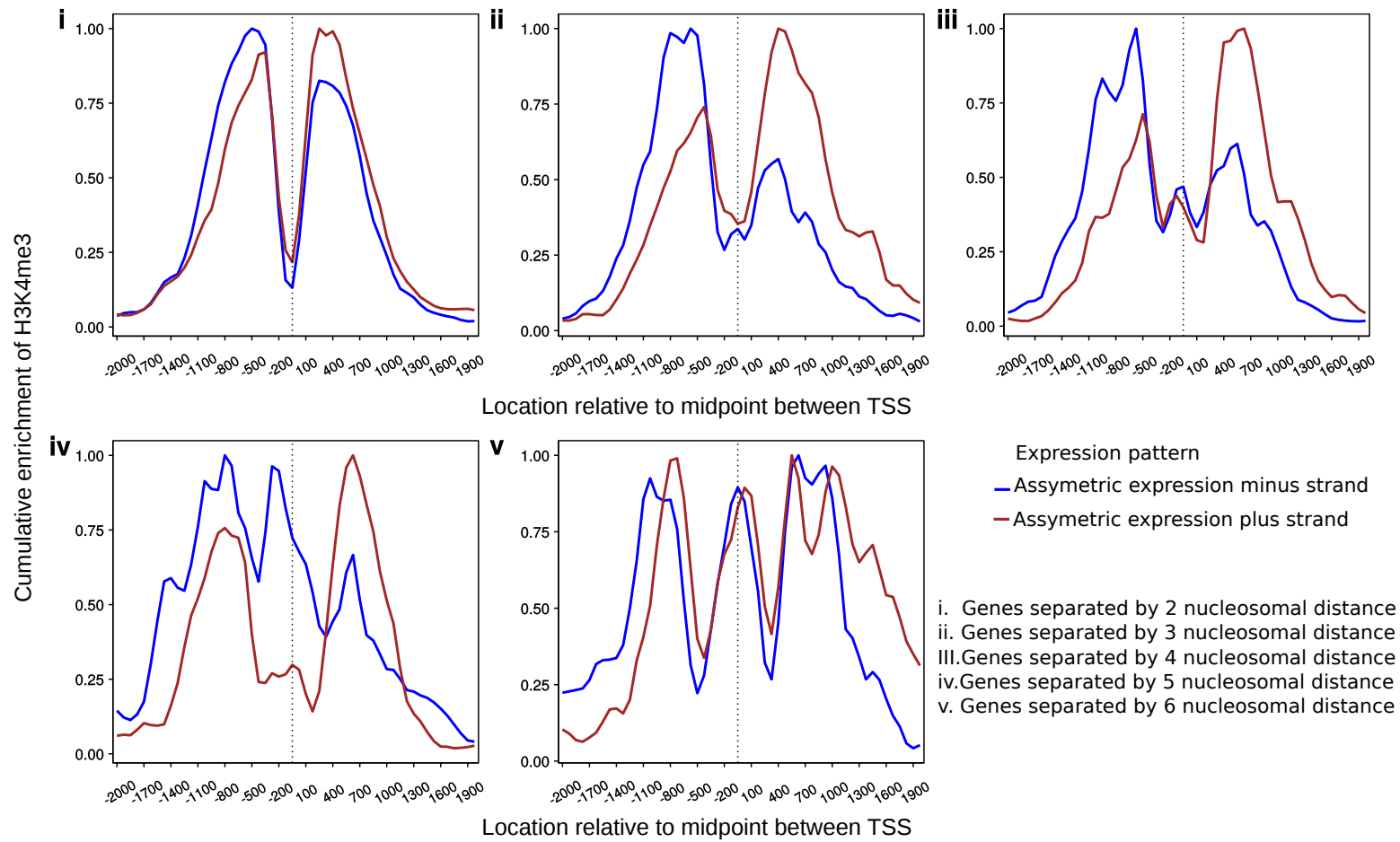

## B

### Symmetrically expressed genes

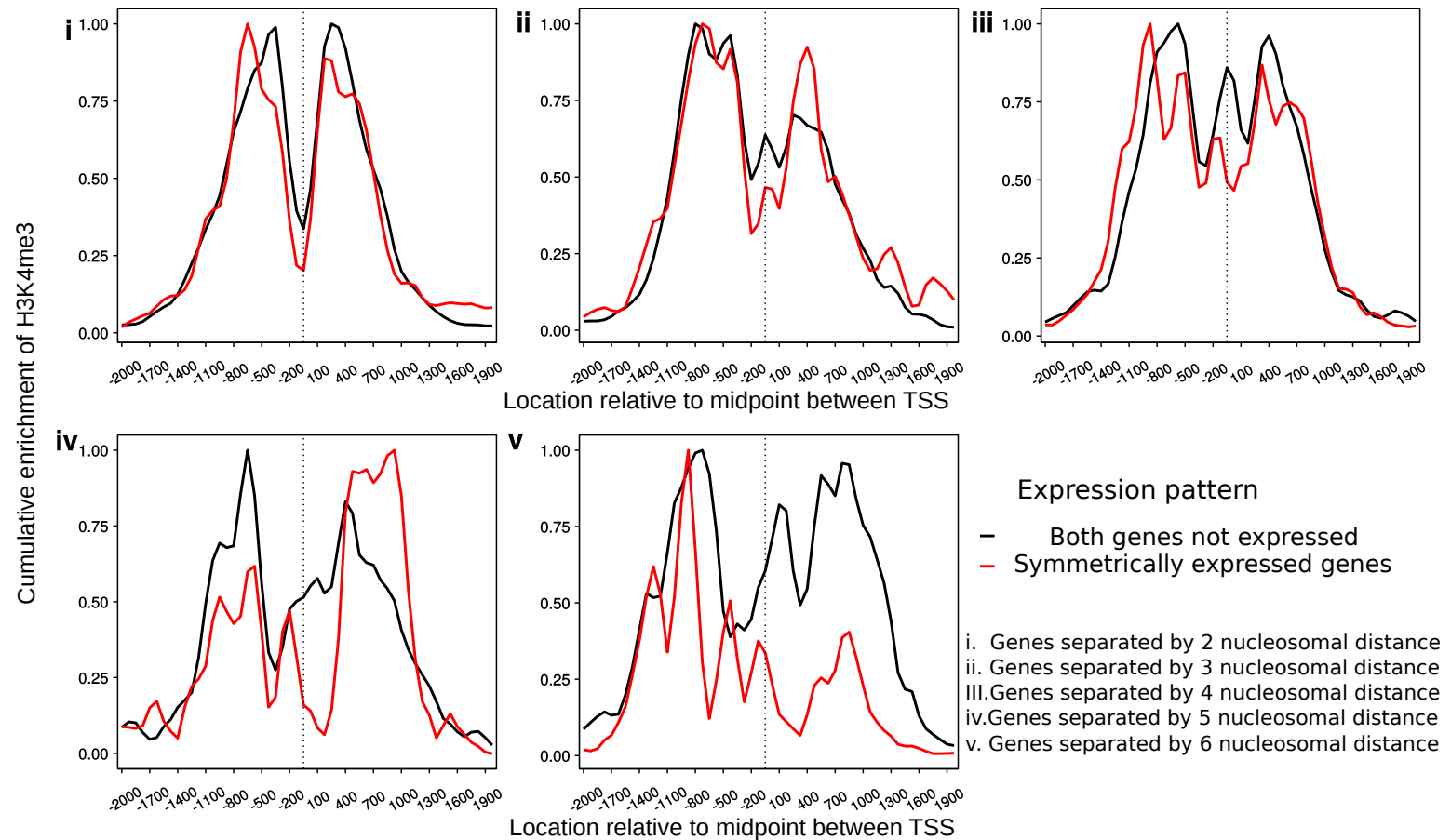

Supplement: Supplementary file 3 — Figure S3. H3K4me3 distribution on bidirectional gene with different intergenic distances in H1 ES cells. The figure shows enrichment of H3K4me3 at the bidirectional genes pairs with intergenic distance upto 1000 bp. Intergenic distance is represented as the number of nucleosomes that could potentially be accommodated. Data are shown for the gene pairs which have intergenic region that could contain 2 to 6 nucleosomes assuming 170 bp length for wrapping around each octamer and inclusive of the 20 bp linker. Cumulative expression is calculated by summation of fold enrichment signal at every location in a 4 KB window for each category and dividing by the highest value of signal in the respective category as described in ‘Methods’. (A) Cumulative enrichment of H3K4me3 on bidirectional genes which are asymmetric with respect to their expression profiles. (B) Cumulative enrichment of H3K4me3 on bidirectional genes whose expression profiles are symmetric. (PDF 347 kb) [file 12864_2018_4697_MOESM3_ESM.pdf]

# Figure S4

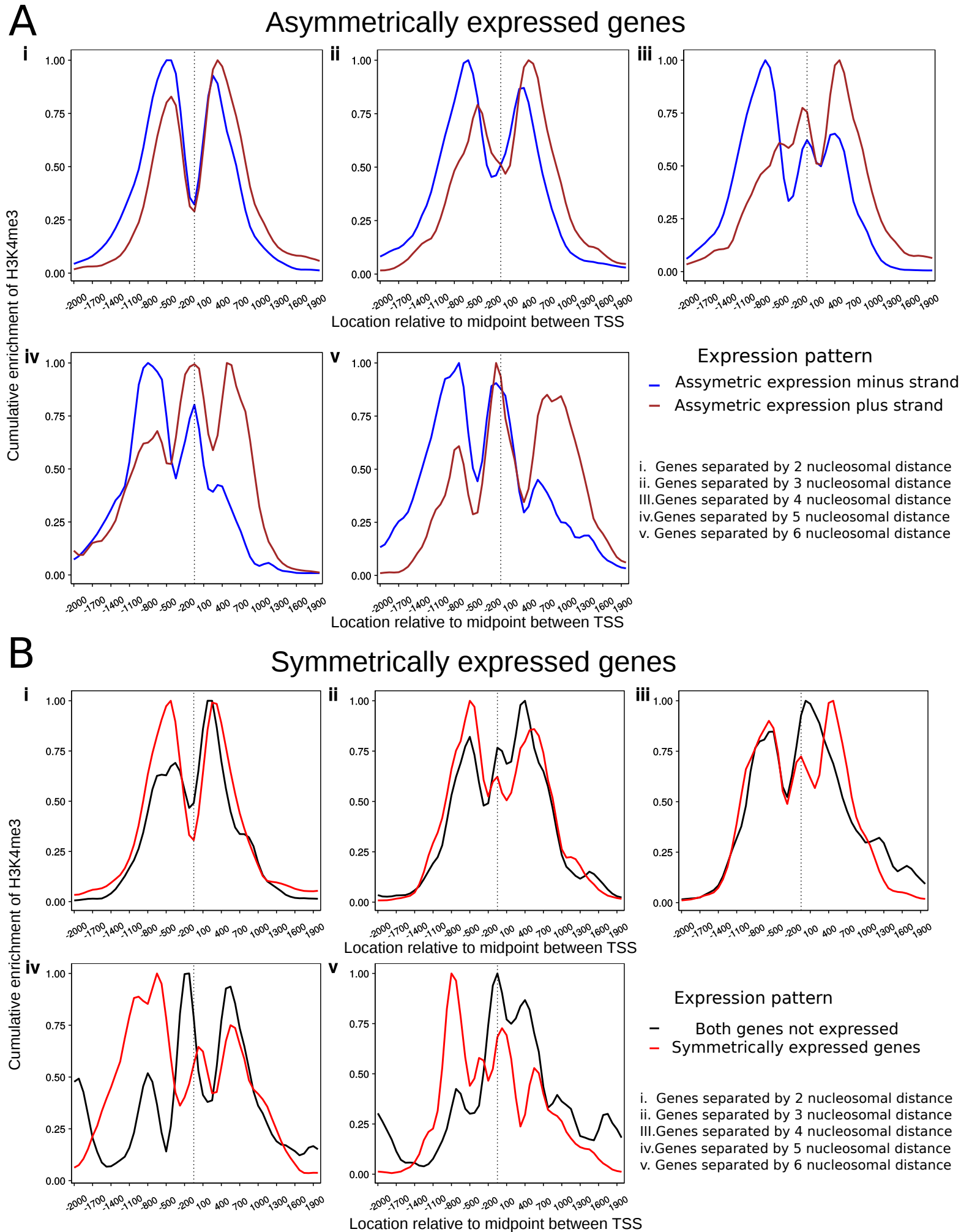

Supplement: Supplementary file 4 — Figure S4. H3K4me3 distribution on bidirectional gene with different intergenic region in CD4+ T cells. The figure shows enrichment of H3K4me3 at the bidirectional genes pairs with intergenic distance up to 1000 bp. Intergenic distance is represented as the number of nucleosomes that could potentially be accommodated. Data are shown for the gene pairs which have intergenic region that could contain 2 to 6 nucleosomes assuming 170 bp length for wrapping around each octamer and inclusive of the 20 bp linker. Cumulative expression is calculated by summation of fold enrichment signal at every location in a 4 Kb window for each category and dividing by the highest value of signal in the respective category as described in ‘Methods’. (A) Cumulative enrichment of H3K4me3 on bidirectional genes which are asymmetric with respect to their expression. (B) Cumulative enrichment of H3K4me3 on bidirectional genes which are symmetric with respect to their expression. (PDF 1048 kb) [file 12864_2018_4697_MOESM4_ESM.pdf]

# Figure S5

## A

### Asymmetrically expressed genes

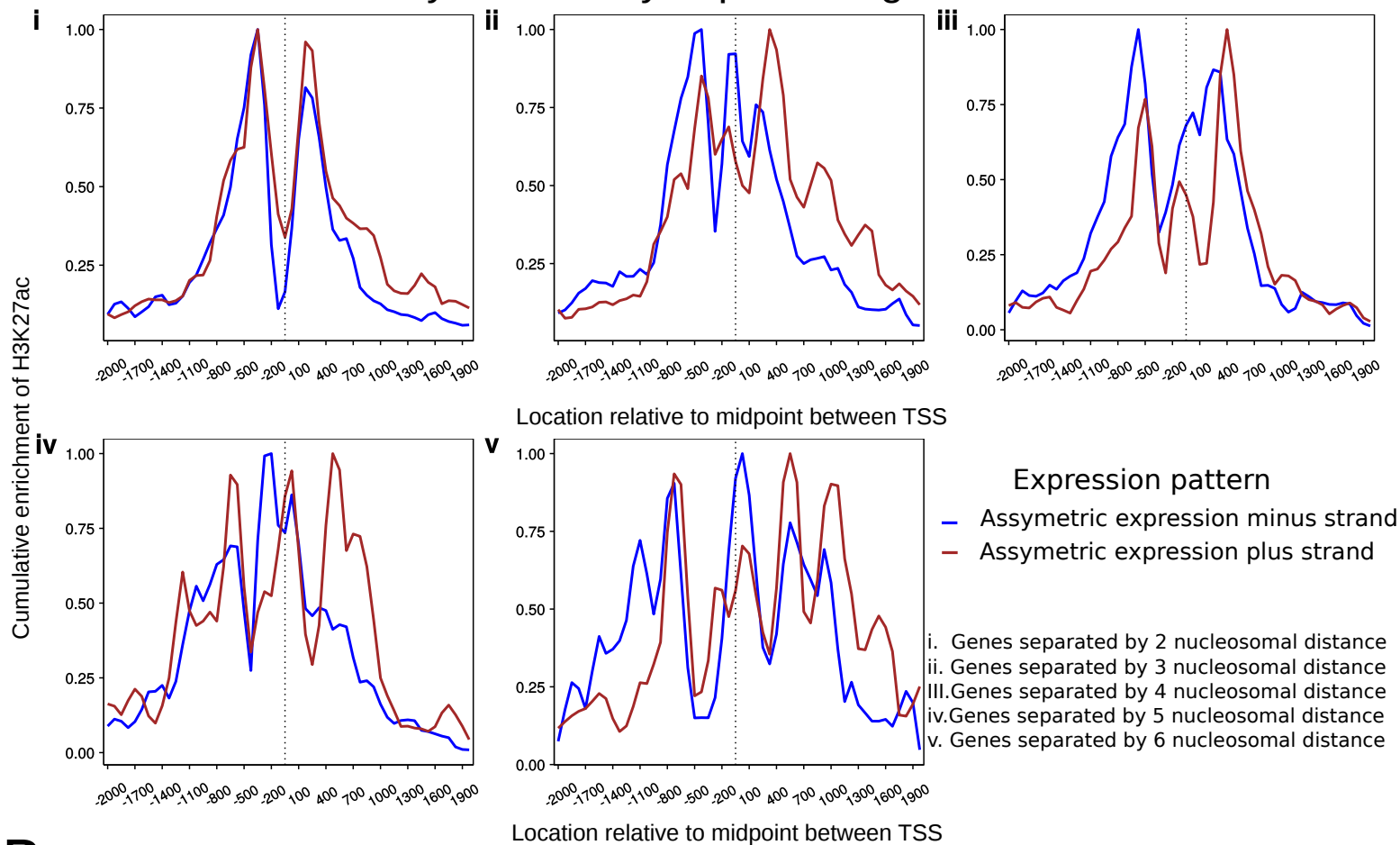

## B

### Symmetrically expressed genes

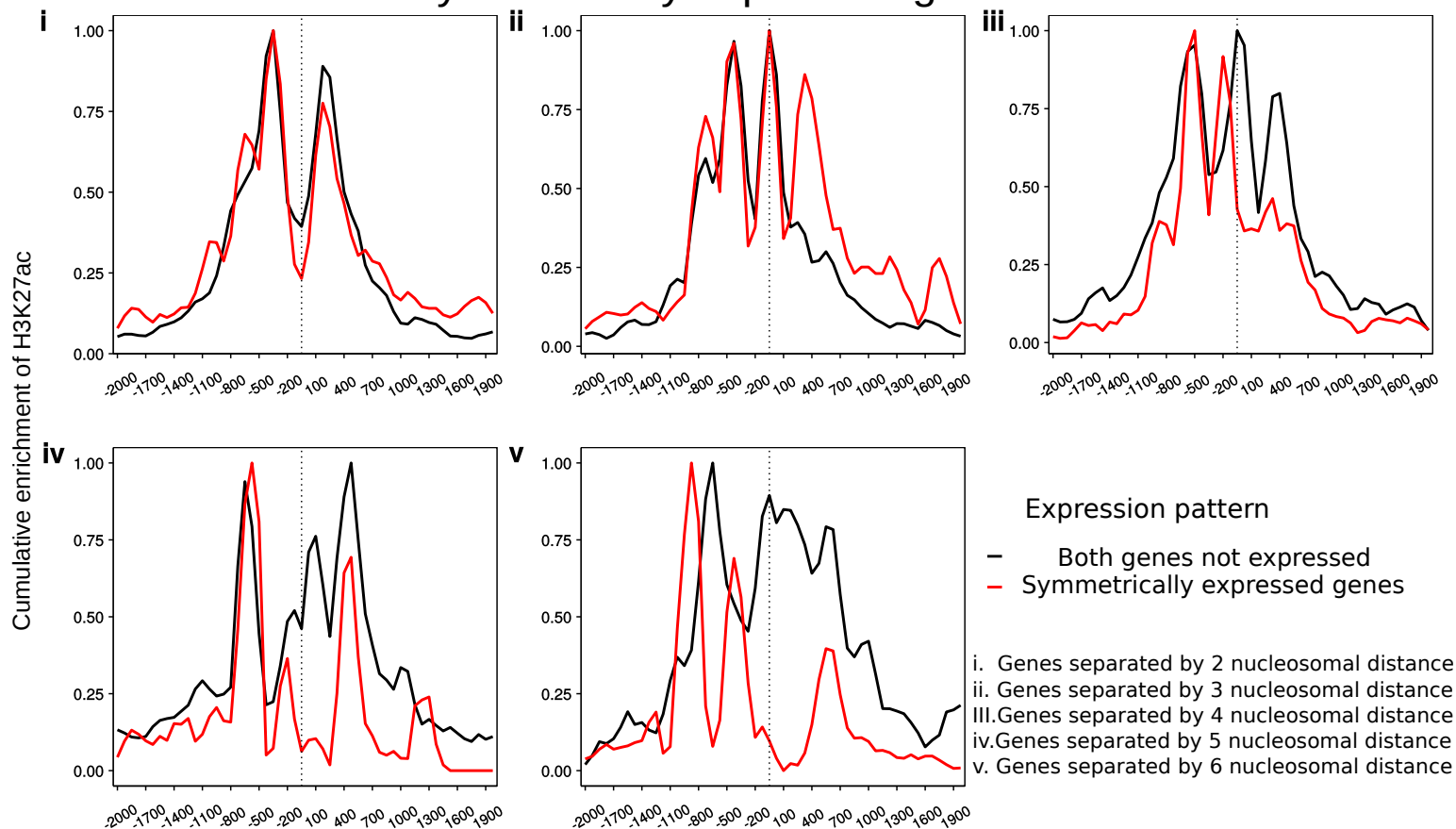

Supplement: Supplementary file 5 — Figure S5. H3K27ac distribution on bidirectional gene with different intergenic region in H1 ES cells. The figure shows enrichment of H3K27ac at the bidirectional genes pairs with intergenic distance up to 1000 bp. Intergenic distance is represented as the number of nucleosomes that could potentially be accommodated. Data are shown for the gene pairs which have intergenic region that could contain 2 to 6 nucleosomes assuming 170 bp length for wrapping around each octamer and inclusive of the 20 bp linker. Cumulative expression is calculated by summation of fold enrichment signal at every location in a 4 Kb window for each category and dividing by the highest value of signal in the respective category as described in ‘Methods’. (A) Cumulative enrichment of H3K27ac on bidirectional genes which are asymmetric with respect to their expression profiles. (B) Cumulative enrichment of H3K27ac on bidirectional genes which are symmetric with respect to their expression profiles. (PDF 1135 kb) [file 12864_2018_4697_MOESM5_ESM.pdf]

# Figure S6

## A

### Asymmetrically expressed genes

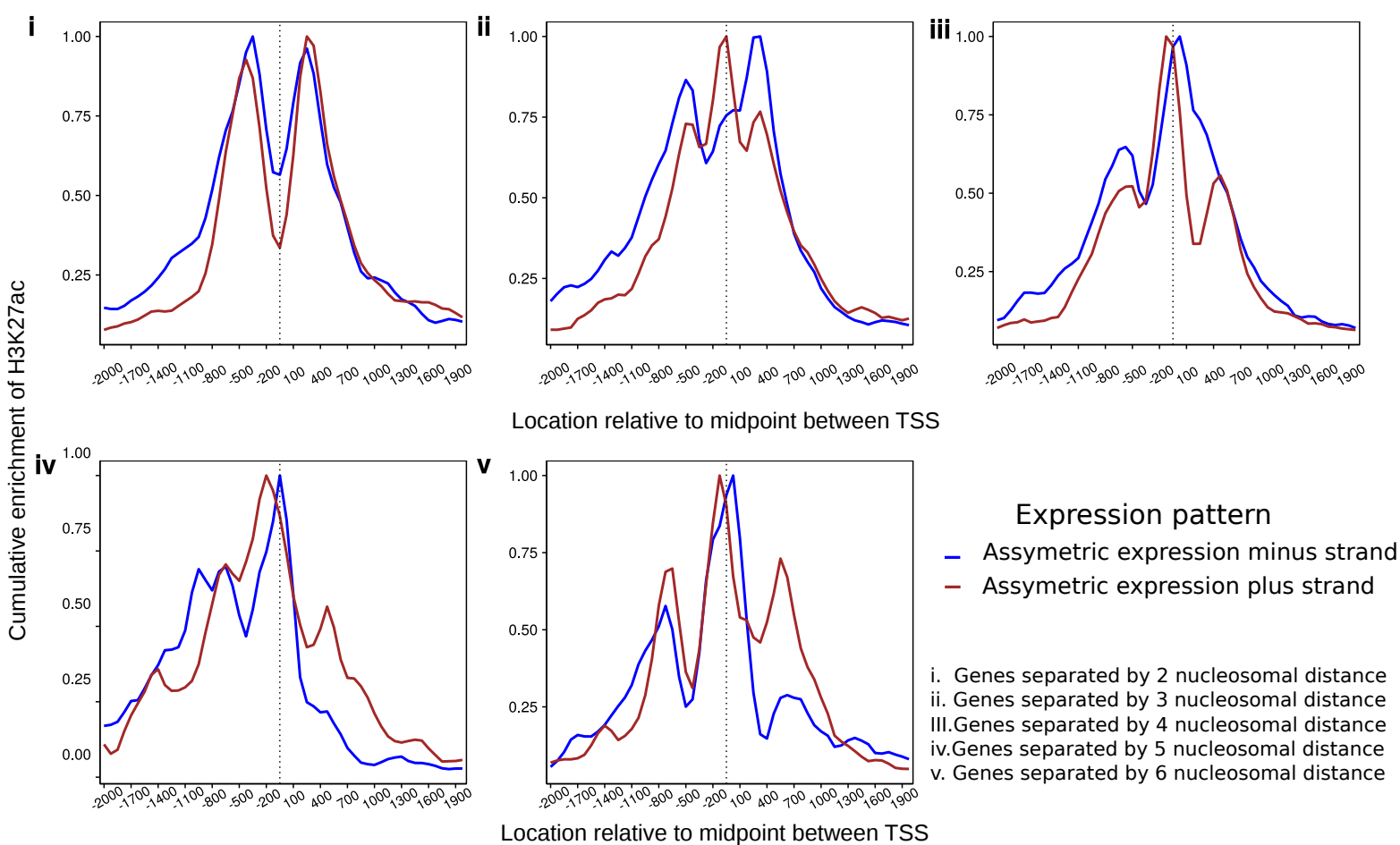

## B

### Symmetrically expressed genes

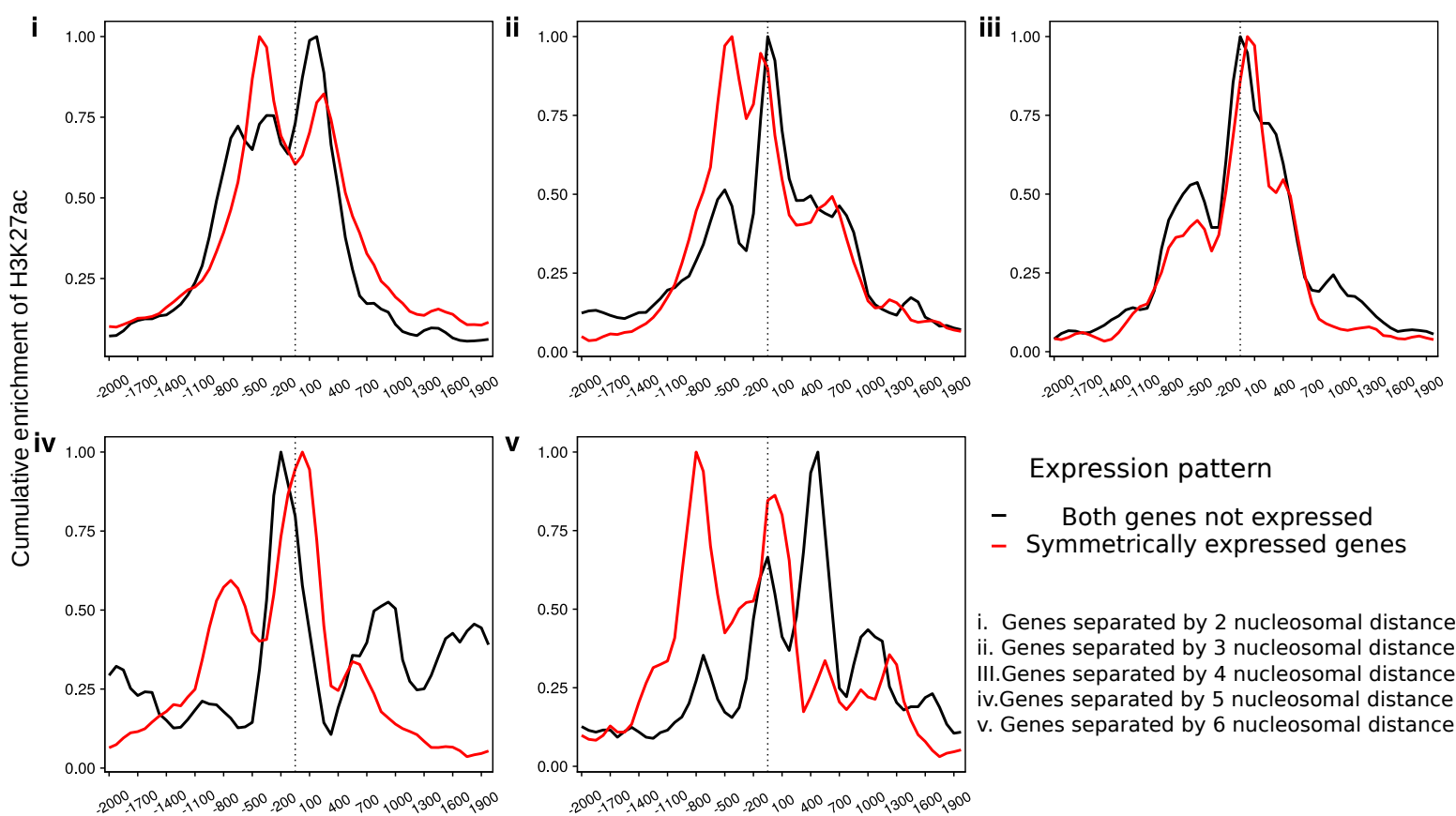

Supplement: Supplementary file 6 — Figure S6. H3K27ac distribution on bidirectional gene with different intergenic region in CD4 T cells. The figure shows enrichment of H3K27ac at the bidirectional genes pairs with intergenic distance up to 1000 bp. Intergenic distance is represented as the number of nucleosomes that could potentially be accommodated. Data are shown for the gene pairs which have intergenic region that could contain 2 to 6 nucleosomes assuming 170 bp length for wrapping around each octamer and inclusive of 20 bp linker. Cumulative expression is calculated by summation of fold enrichment signal at every location in a 4 Kb window for each category and dividing by the highest value of signal in the respective category as described in ‘Methods’. (A) Cumulative enrichment of H3K27ac on bidirectional genes which are asymmetric with respect to their expression profiles. (B) Cumulative enrichment of H3K27ac on bidirectional genes which are symmetric with respect to their expression profiles. (PDF 819 kb) [file 12864_2018_4697_MOESM6_ESM.pdf]

# Figure S7

## A

### Asymmetrically expressed genes

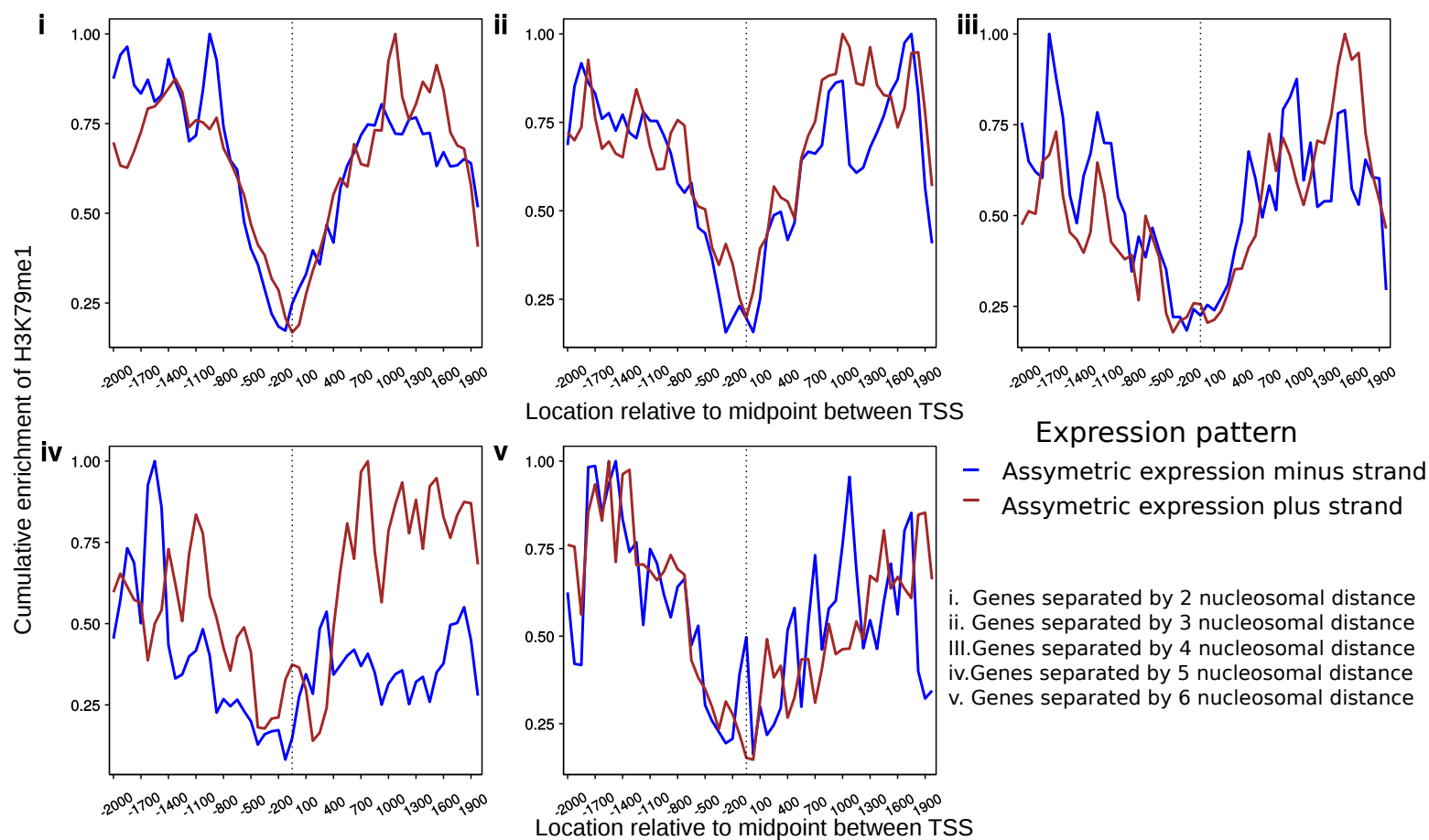

## B

### Symmetrically expressed genes

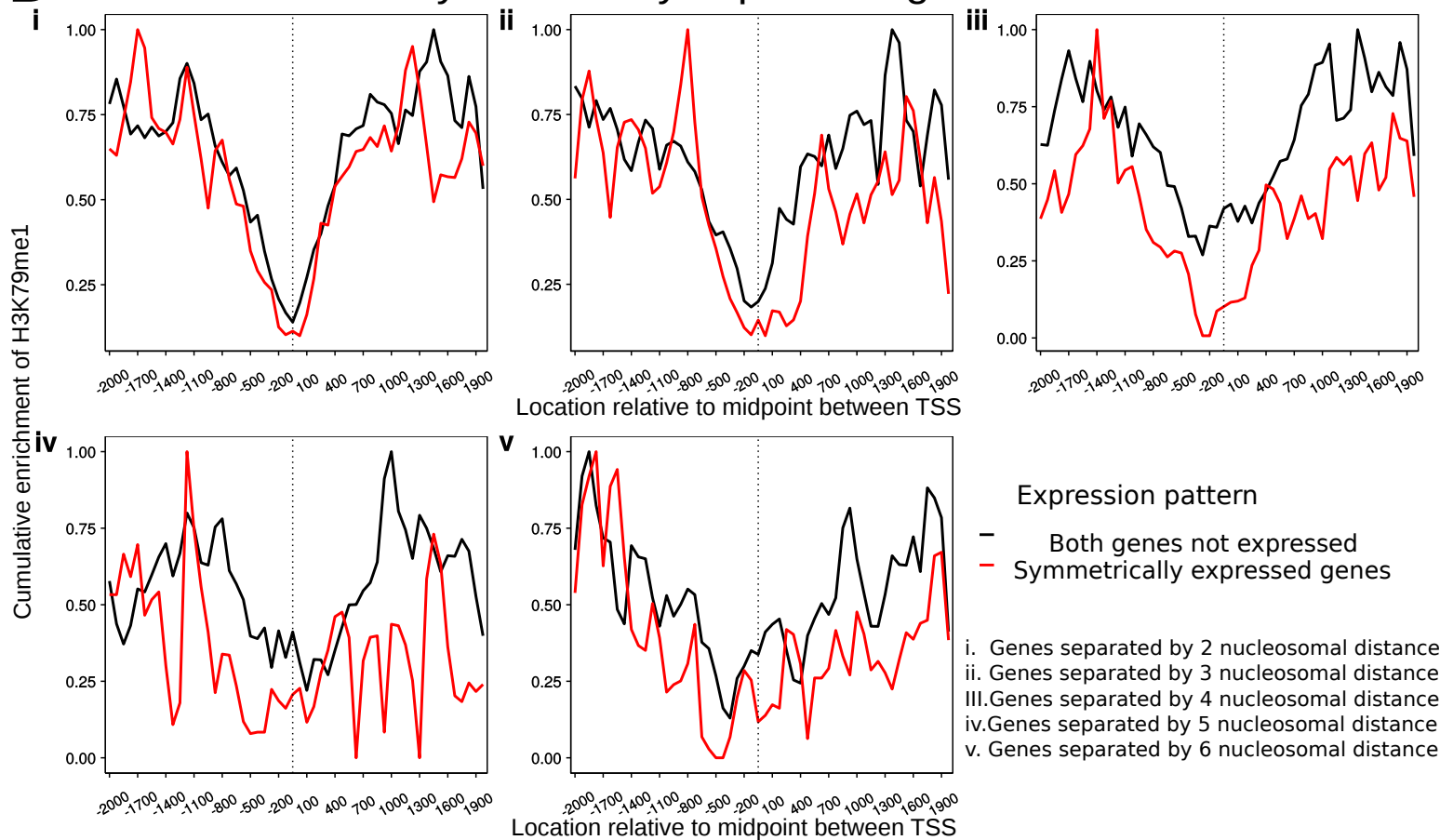

Supplement: Supplementary file 7 — Figure S7. H3K79me1 distribution on bidirectional gene with different intergenic region in H1 ES cells. The figure shows enrichment of H3K79me1 at the bidirectional genes pairs with intergenic distance up to 1000 bp. Intergenic distance is represented as the number of nucleosomes that could potentially be accommodated. Data are shown for the gene pairs which have intergenic region that could contain 2 to 6 nucleosomes assuming 170 bp length for wrapping around each octamer and inclusive of 20 bp linker. Cumulative expression is calculated by summation of fold enrichment signal at every location in a 4 KB window for each category and dividing by the highest value of signal in the respective category as described in ‘Methods’. (A) Cumulative enrichment of H3K79me1 on bidirectional genes which are asymmetric with respect to their expression profiles. (B) Cumulative enrichment of H3K79me1 on bidirectional genes which are symmetric with respect to their expression profiles. (PDF 831 kb) [file 12864_2018_4697_MOESM7_ESM.pdf]

# Figure S8

## A

### Asymmetrically expressed genes

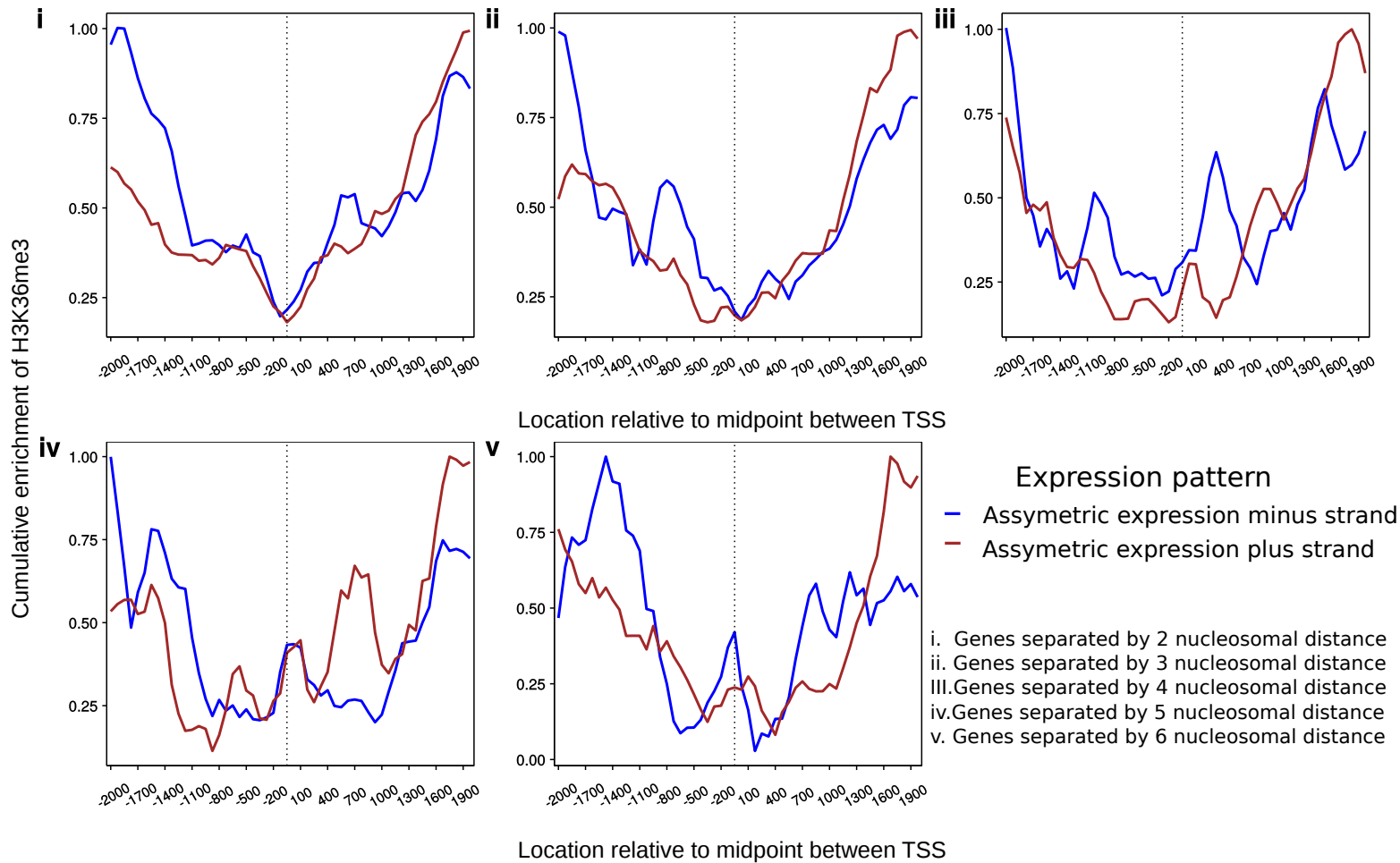

## B

### Symmetrically expressed genes

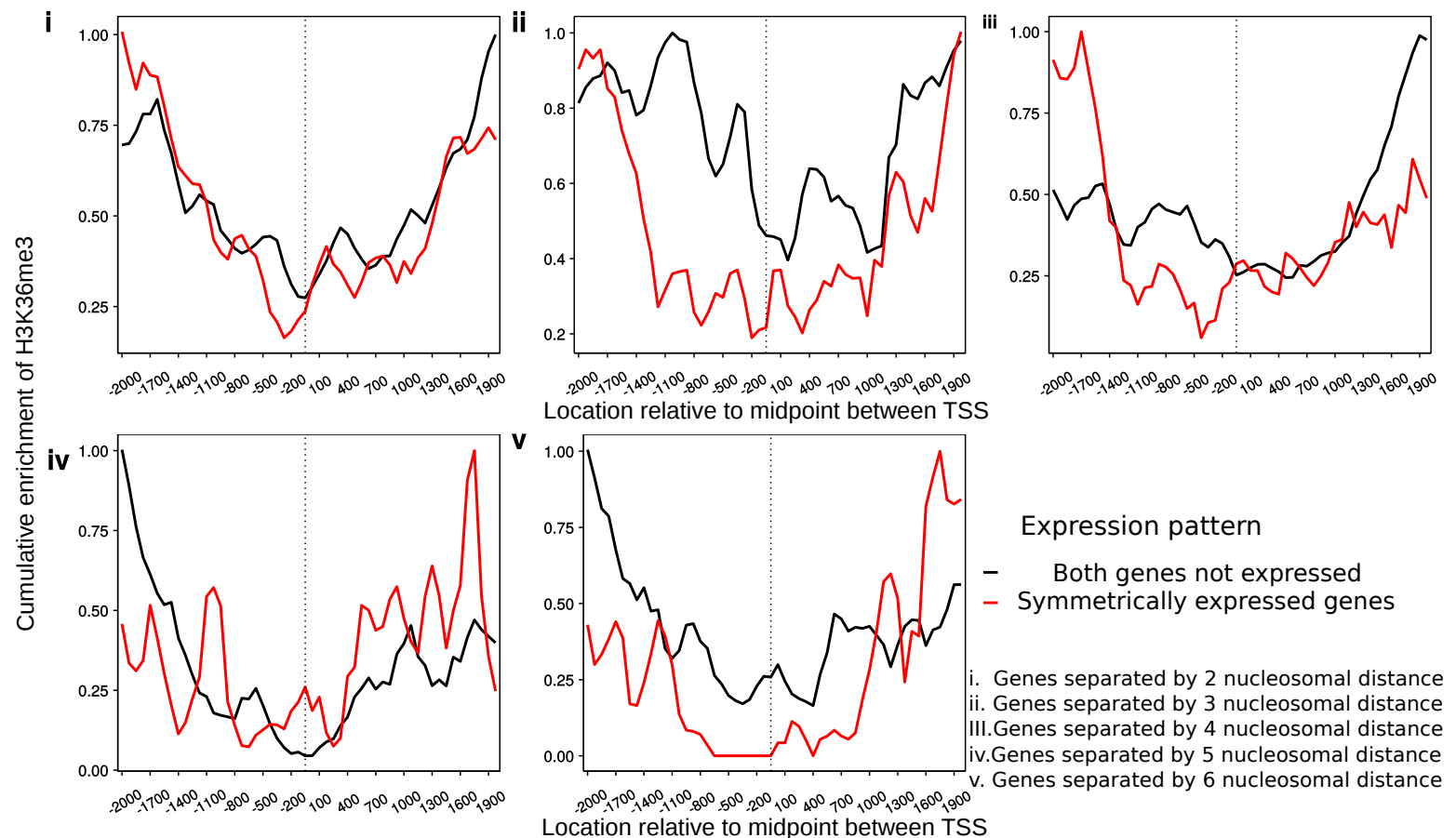

Supplement: Supplementary file 8 — Figure S8. H3K36me3 distribution on bidirectional gene with different intergenic region in H1 ES cells. The figure shows enrichment of H3K79me1 at the bidirectional genes pairs with intergenic distance up to 1000 bp. Intergenic distance is represented as the number of nucleosomes that could potentially be accommodated. Data are shown for the gene pairs which have intergenic region that could contain 2 to 6 nucleosomes assuming 170 bp length for wrapping around each octamer and inclusive of 20 bp linker. Cumulative expression is calculated by summation of fold enrichment signal at every location in a 4 KB window for each category and dividing by the highest value of signal in the respective category as described in ‘Methods’. (A) Cumulative enrichment of H3K36me3 on bidirectional genes which are asymmetric with respect to their expression profiles. (B) Cumulative enrichment of H3K36me3 on bidirectional genes which are symmetric with respect to their expression profiles. (PDF 829 kb) [file 12864_2018_4697_MOESM8_ESM.pdf]

**Figure S9**

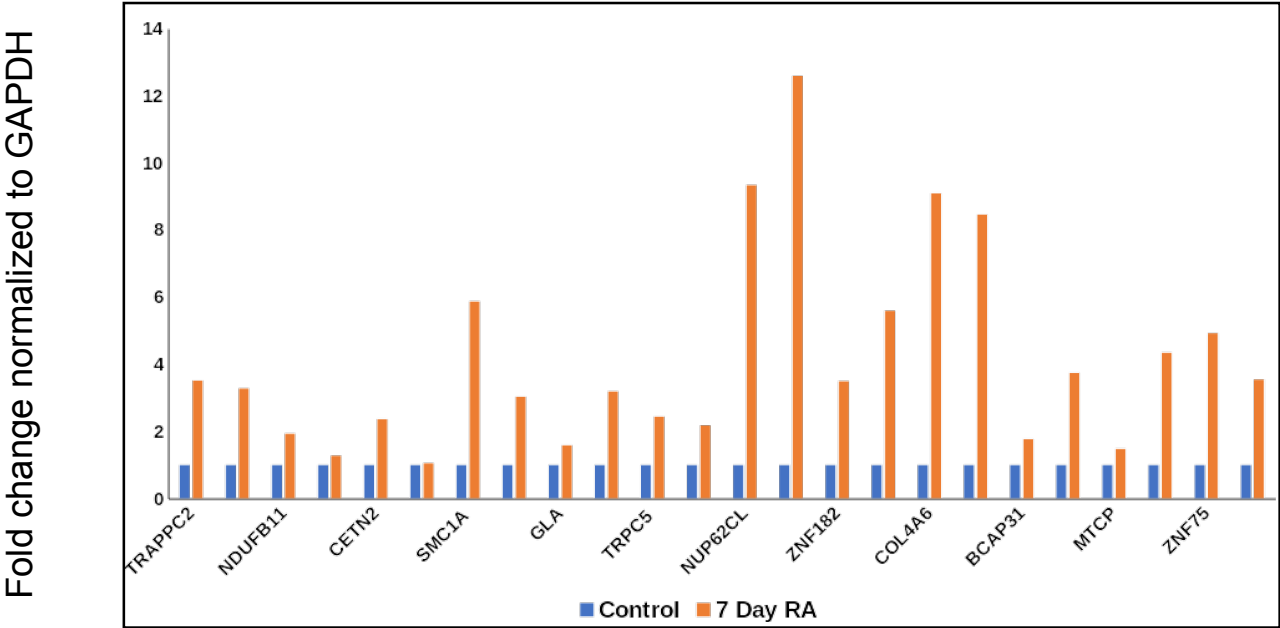

Supplement: Supplementary file 9 — Expression profile 10 bidirectional gene pairs upon RA mediated differentiation of NT2D-1 cells. These gene pairs were tested for expression differences with real-time PCR upon RA mediated differentiation as described in Methods. (PDF 58 kb) [file 12864_2018_4697_MOESM9_ESM.pdf]
